# Role of the mechanisms for antibody repertoire diversification in monoclonal light chain deposition disorders: when a friend becomes foe

**DOI:** 10.3389/fimmu.2023.1203425

**Published:** 2023-07-13

**Authors:** Luis Del Pozo-Yauner, Guillermo A. Herrera, Julio I. Perez Carreon, Elba A. Turbat-Herrera, Francisco J. Rodriguez-Alvarez, Robin A. Ruiz Zamora

**Affiliations:** ^1^ Department of Pathology, University of South Alabama-College of Medicine, Mobile, AL, United States; ^2^ Instituto Nacional de Medicina Genomica (INMEGEN), Ciudad de México, Mexico; ^3^ Mitchell Cancer Institute, University of South Alabama-College of Medicine, Mobile, AL, United States

**Keywords:** light chain (AL) amyloidosis, somatic hypermutation, V(D)J rearrangement, protein aggregation, amyloid, immune system, antibodies

## Abstract

The adaptive immune system of jawed vertebrates generates a highly diverse repertoire of antibodies to meet the antigenic challenges of a constantly evolving biological ecosystem. Most of the diversity is generated by two mechanisms: V(D)J gene recombination and somatic hypermutation (SHM). SHM introduces changes in the variable domain of antibodies, mostly in the regions that form the paratope, yielding antibodies with higher antigen binding affinity. However, antigen recognition is only possible if the antibody folds into a stable functional conformation. Therefore, a key force determining the survival of B cell clones undergoing somatic hypermutation is the ability of the mutated heavy and light chains to efficiently fold and assemble into a functional antibody. The antibody is the structural context where the selection of the somatic mutations occurs, and where both the heavy and light chains benefit from protective mechanisms that counteract the potentially deleterious impact of the changes. However, in patients with monoclonal gammopathies, the proliferating plasma cell clone may overproduce the light chain, which is then secreted into the bloodstream. This places the light chain out of the protective context provided by the quaternary structure of the antibody, increasing the risk of misfolding and aggregation due to destabilizing somatic mutations. Light chain-derived (AL) amyloidosis, light chain deposition disease (LCDD), Fanconi syndrome, and myeloma (cast) nephropathy are a diverse group of diseases derived from the pathologic aggregation of light chains, in which somatic mutations are recognized to play a role. In this review, we address the mechanisms by which somatic mutations promote the misfolding and pathological aggregation of the light chains, with an emphasis on AL amyloidosis. We also analyze the contribution of the variable domain (V_L_) gene segments and somatic mutations on light chain cytotoxicity, organ tropism, and structure of the AL fibrils. Finally, we analyze the most recent advances in the development of computational algorithms to predict the role of somatic mutations in the cardiotoxicity of amyloidogenic light chains and discuss the challenges and perspectives that this approach faces.

## 1 Introduction

One factor that influenced the evolution of complex multicellular organisms such as humans is the dual nature of the interaction with bacteria, fungi, viruses, and other microorganisms that coevolved with them, some of which colonized the multicellular organisms to form what is called microbiota (1, 2). While both the multicellular host and its microbiota benefited from the reciprocal interaction (3, 4), the former must avoid succumbing due to the uncontrolled invasion of the later (1, 2). Hence, different strategies and molecular mechanisms evolved in the multicellular organisms to detect and kill pathogenic microbes and parasites (host defend), while maintaining microbiota homeostasis, that is, a balanced interaction with those non-pathogenic microbes that are beneficial (mutualism) (5, 6).

Regardless of their complexity, all mechanisms evolved to protect Metazoans against invading microbes operate on the same biological principle: before any response can be elicited, recognition of an external signal must be achieved (7). The recognition of the external signal, essentially a non-self-molecule, is accomplished by a cell receptor, triggering a cascade of multiple converging events whose ultimate goal is the removal of the “signal” that triggered the response. Plants, which lack a circulatory system and mobile immune cells, evolved an innate immune system that is broadly divided into pathogen-associated molecular pattern- or PAMP-triggered immunity (PTI), the first layer of the immune response, and effector-triggered immunity (ETI). PTI is activated upon perception of molecules with conserved motifs derived from pathogens by surface membrane-anchored pattern recognition receptors (PRRs) (8, 9). Invertebrates also relay on innate defense mechanisms, which use a large diversity of molecules with broad-spectrum bactericidal and fungicidal properties to defend themselves from microbes (7). The evolutionary emergence of vertebrates, about 500 million years ago, was accompanied by a new form of immune protection, the adaptive immune system (10, 11). In vertebrates, the sophisticated but relatively nonspecific innate immunity cooperates with the highly refined adaptive immune system to elicit a highly effective protective response characterized by antigen specificity and immune memory (10). The adaptive immune system uses highly diversified repertoires of antigen receptors located in the membrane of immunocompetent cells, which, remarkably, are structurally different in jawed and jawless vertebrates. In jawless vertebrates, the adaptive immune receptors are termed variable lymphocyte receptors (VLRs) and are constructed from leucine-rich repeat modules. In contrast, the adaptive immune system in jawed vertebrates uses structurally similar antigen receptors, named immunoglobulins and T cell receptors (TCRs), clonally expressed by B and T lymphocytes, respectively (10, 12). Such difference between the adaptive immune receptors in jawless and jaw vertebrates is a consequence of having evolved independently in both groups (7, 10, 12). Immunoglobulins, also known as antibodies, and TCR are generated through combinatorial recombination of gene segments, a process known as V(D)J gene recombination, which theoretically can generate an almost infinite number of antigen-binding specificities. B and T lymphocytes displaying these membrane antigen receptors travel through the circulatory system to detect pathogens or mutated cells. The recognition of a foreign molecule by B and T lymphocytes leads the clonal expansion, which, in the case of B-cell lymphocytes, is accompanied by somatic hypermutation (SHM). This molecular event, unique to B cells, introduces mutations in the variable regions of antibodies that increase the binding affinity for the antigen, greatly expanding the antibody repertoire (13). The end of the road for B lymphocytes activated by antigens is differentiation into plasma cells, which do not display membrane-bound antibodies, but actively secrete them in a soluble form. Antibodies secreted by plasma cells act as mediators of the humoral immune response, promoting different mechanisms of inactivation and/or elimination of foreign molecules and invading pathogenic microbes (10). SHM plays a key role in the antibody response, as it transforms germline-encoded antibodies characterized by low affinity and propensity to cross-react with other antigens, into a more specific antibody with a higher binding affinity (6, 13). While this is key for mounting an effective antibody response, SHM may also be a contributor to the pathogenesis of several human diseases, such as when it generates antibodies that recognize self-antigens (14), or when it promotes misfolding and aggregation of the immunoglobulin light chain (LC) (15, 16). This review focuses on the role of the mechanism for diversification of the human repertoire of antibodies in the molecular pathogenesis of the group of diseases related to misfolding and deposition of monoclonal light chains with an emphasis on light chain-derived (AL) amyloidosis.

## 2 Structural and genetic basis of antibody function

### 2.1 Structural bases of antibody function

**Figure 1:**
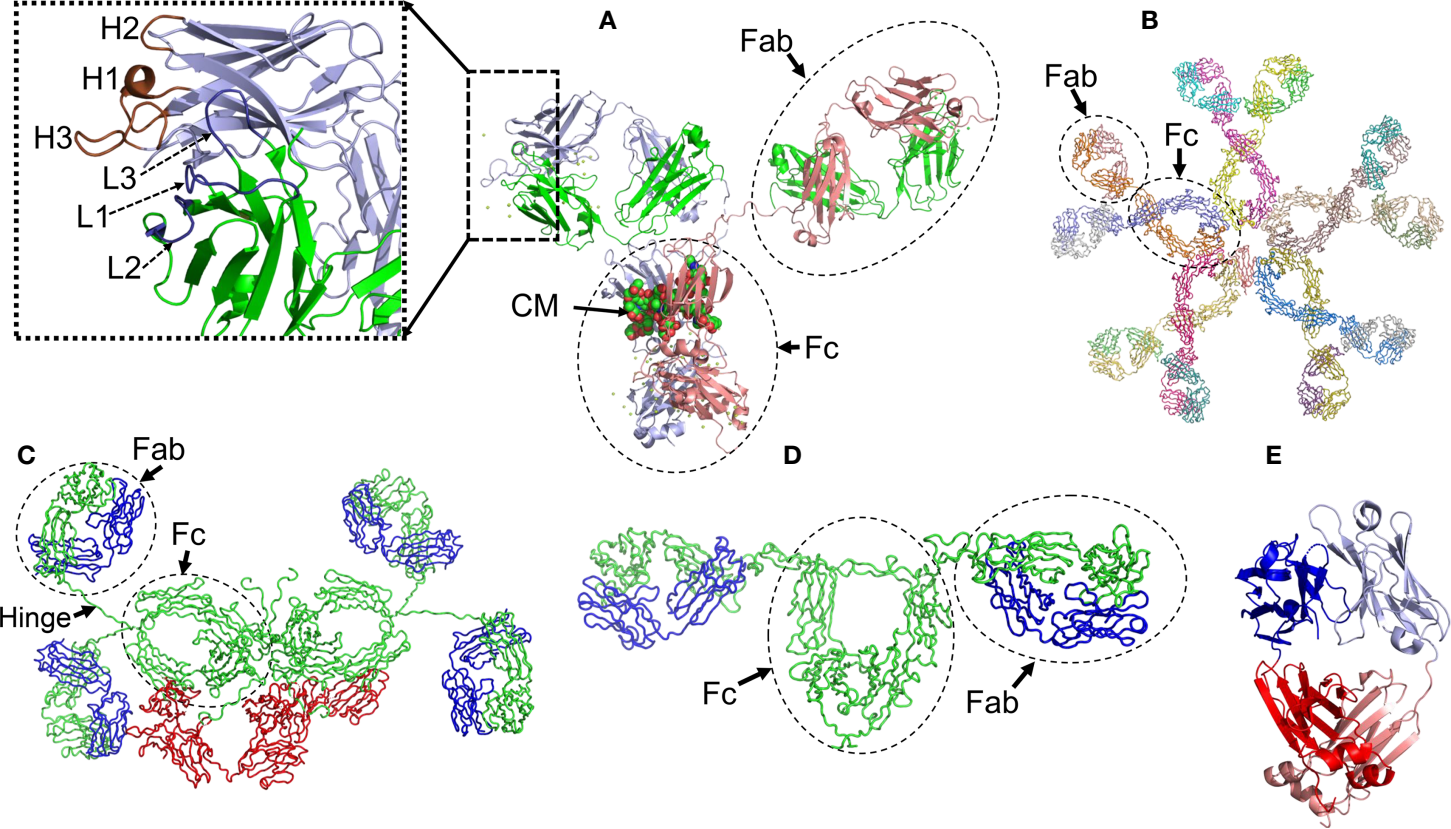
Structural characteristics of human immunoglobulins. **(A)** Crystal structure of the intact human IgG B12 with broad and potent activity against primary HIV-1 isolates (Method: X-ray diffraction - PDB 1HZH). **(B)** Solution structure of human Immunoglobulin M (Method: Solution X-ray scattering – PDB 2RCJ) **(C)** Solution structure of human secretory IgA1 (Method: Solution X-ray scattering – PDB 3CHN). **(D)** Semi-extended solution structure of human myeloma immunoglobulin D (Method: solution X-ray scattering – PDB 1ZVO). **(E)** Crystal structure of amyloidogenic and cardiotoxic Bence-Jones Λ2 LC dimer H9 (Method: X-ray crystallography – PDB 5M6A). The insert in A shows the spatial conformation of the light chain (L1 to L3, colored in deep blue) and heavy chain (H1 to H3, colored in brown) CDRs. CM stands for carbohydrate moiety attached to the C_H2_ domain. The Fab (fragment antigen-binding) and Fc (fragment crystallizing) regions of each antibody are indicated. In C, the secretory component of the dimeric IgA is shown in red. The long hinge connecting the Fab and Fc regions in IgA1 antibodies is also indicated. Due to limitations of the structural method applied, the spatial location of the J chain was not determined. In C and D, the light and heavy chains are shown in blue and green, respectively. In E, the variable domains of the light chain dimer are shown in blue and light blue, while the constant domains are shown in red and salmon. Note that the Bence Jones dimer is stabilized by a LC-LC interface that mimics the LC-HC interface of a functional antibody. All structures were prepared with PyMOL (PyMOL Molecular Graphics System, Version 2.5.2, Schrödinger, LLC).

Antibodies are glycoproteins with a basic structural unit formed by the association of two heavy (HC) and two LCs (**Figure 1A**). The human genome encodes for five major HC isotypes; γ, δ, α, ε, and µ, which determine the antibody classes IgG, IgD, IgA, IgE, and IgM, respectively. HCs γ, α and δ are ∼450 amino acids long and fold into four domains, the N-terminal variable domain (V_H_) and three constant domains, designated C_H1_ to C_H3_, arranged in tandem. HCs µ and ε are ∼100 residues longer and fold into one additional constant domain (C_H4_) (**Figures 1A–D**). Humans produce two different types of LCs, kappa (κ) and lambda (Λ), which are ∼215-225 amino acids long and fold into two domains, the N-terminal variable (V_L_) and the C-terminal constant (C_L_) domains (**Figure 1E**) (17).

IgG and IgA antibodies are subdivided into four (IgG1, IgG2, IgG3, and IgG4) and two (IgA1 and IgA2) subclasses, respectively, which reflects the number of γ (γ1 to γ4) and α (α1 and α2) HC genes (isotypes) in the human genome (18, 19). Although the IgG subclasses share ∼90% identity in amino acid sequence, they differ in several biological properties, such as immune complex formation, complement activation, ability to trigger effector cells response, half-life, and placental transport (18). IgA subclasses also share high sequence identity, differing from each other mainly in the structure of their hinge region, located between Val222 and Cys241, and in the number of glycosylation sites. The hinge region of human IgA1 antibodies spans 18 amino acids and contains up to five O-linked glycans on serine and threonine residues. In contrast, IgA2 presents a 5 amino acid hinge and lacks O-linked glycans (20). Structural differences between IgA subclasses translate into distinct effector functions in immune cells, as well as susceptibility to inactivation by cleavage at hinge regions by proteases produced by some pathogenic bacteria (21). Moreover, IgA subclasses are not expressed equal in body fluids, since IgA1 makes up around 80-90% of total serum IgA, but in mucosal surfaces, both isotypes are more evenly distributed (19, 22).

Antibodies of the IgG, IgD, and IgE classes are produced only as monomers of the basic unit, with molecular weight (MW) of ∼150kDa for both IgG and IgD, and 180kDa for IgE. In contrast, IgA antibodies are produced in two formats, as monomers with MW of ∼150kDa, and dimers of ∼385kDa. Monomeric IgA is secreted into the bloodstream, while the dimeric form, known as secretory IgA (SIgA), is secreted to the mucous membranes of the respiratory, genitourinary, and digestive tracks, where it contributes to mucosal immunity and preserving the microbiota homeostasis (6, 22). The dimeric complex of SIgA contains two additional peptides, termed the J-chain and secretory component (23) (**Figure 1C**). IgM antibodies, on the other hand, are secreted mostly in a pentameric configuration with a MW of 900 kDa (24) (**Figure 1**). The quaternary structure of antibodies is stabilized by interchain disulfide bonds that link each HC-LC pair and one HC to another, as well as by a myriad of weak non-covalent interactions between HC and LC residues located at the V_H_-V_L_ and C_H1_-C_L_ interfaces (25) (**Figure 1**). Contacts at the LC-HC interface contribute critically to the proper folding of the antibodies in the B lymphocyte. This is particularly relevant for the C_H_1 domain, which folds *via* a template-assisted mechanism that requires interactions with key residues in the dimerization interface with a fully folded C_L_ domain (26). The chaperone role of the LC in HC folding is considered part of the control mechanisms in B cells to ensure correct folding and transport of antibodies (26). As will be discussed in more detail later in this review, some hematological disorders are characterized by the presence of a monoclonal LC circulating in the blood in a free state. In this circumstance, the monoclonal LC tends to form dimers that can be detected in serum and urine (**Figure 1E**).

Both the variable and constant domains of antibodies exhibit the β-sandwich fold, a distinctive trait of the immunoglobulins. The immunoglobulin fold consists of two antiparallel β sheets with a Greek key topology, composed of 4-5 strands, which associate face‐to‐face to form a two-layer sandwich (**Figure 1**). The core of the sandwich mostly comprises the side chains of non-polar residues, structured around a highly conserved tryptophan residue that tightly packs against the intradomain disulfide bond. The strands of the β sandwich are connected by loops of variable length. In both the V_H_ and V_L_, three of the connecting loops display high sequence variability and are called Complementarity Determining Region 1 to 3 or CDR1 to CDR3. The regions between the CDRs are called Framework Regions (FRs). The CDRs are spatially oriented in such a way that they form the antibody surface, called the paratope, through which antigen recognition takes place (**Figure 1A**). The conformational properties of the CDRs, and therefore the nature of their interaction with the antigen, are mainly determined by their sequence but are also influenced by short- and long-distant interactions between the CDRs and other segments of the β-sandwich. This means that mutations in the CDRs but also in the FRs may modulate the affinity and specificity of the antigen-antibody interaction. The antigen-antibody interaction relies on spatial complementarity between paratope and epitope, being stabilized by several types of non-covalent forces: hydrophobic interactions, H-bonds, van der Waals forces, and saline bridge (27, 28). Interfacial water molecules may also be involved in H-bonds, bringing residues at the antigen-antibody interface, and therefore contributing to the overall stability of the antigen-antibody complexes (29, 30).

### 2.2 Genetics of human antibodies

**Figure 2:**
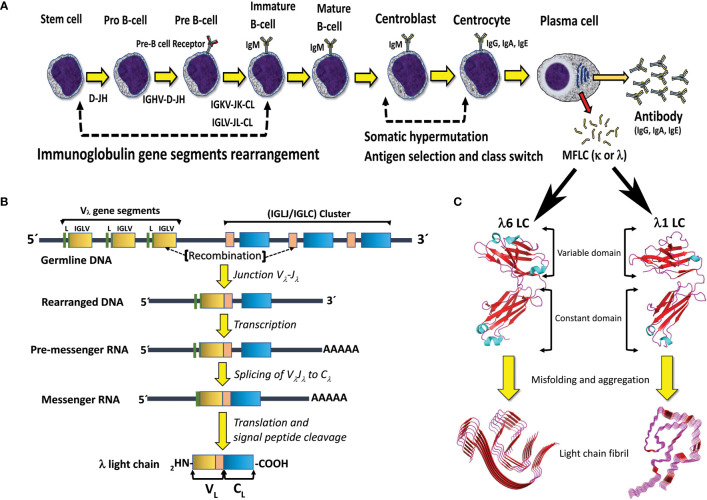
B cell differentiation pathway and mechanisms for generation of antibody diversity. **(A)** The human polyclonal antibody repertoire is generated by two molecular mechanisms: 1) V(D)J gene segments recombination, and 2) somatic hypermutation, which occurs at specific stages of B-cell differentiation. Plasma cells, the final stage of B cell differentiation, secrete large amounts of the specific antibody (IgG, IgA, or IgE). Under certain circumstances, a clone of plasma cells can overproduce the antibody LC and secret it in a free state, which entails the risk of aggregation and disease. MFLC stands for monoclonal free light chain. **(B)** Schematic representation of the IGLV-IGLJ-IGLC gene segment recombination in the Λ LC locus. Note that the V_L_ domain of the LC is encoded by the IGLV and IGLJ gene segments, while the C_L_ domain is encoded by the IGLC gene. **(C)** Structural characteristics of the immunoglobulin LCs and AL amyloid fibrils. The LC structures correspond to the full-length (FL) Λ6 LC of the anti-Hepcidin Fab (PDB 3H0T) and the Λ1 monoclonal free LC (Bence Jones protein) LOCW, both determined by x-ray diffraction. The variable (V_L_) and constant (C_L_) domains are indicated. The regions in β-strand conformation are colored red. The AL fibril structures correspond to the ex-vivo Λ6 (PDB 6HUD) and Λ1 (PDB 6IC3) AL fibrils obtained from the cardiac deposits in patients with AL amyloidosis determined by cryo-EM. The β core of the AL fibrils is composed only of segments of the V_L_ domain. The FL LCs and AL fibrils shown in the figure are not related to each other. They were included in the same figure only for comparative purposes. Structures shown in C were prepared with PyMOL (PyMOL Molecular Graphics System, Version 2.5.2, Schrödinger, LLC).

Functional HC and LC genes are assembled by the combinatorial recombination of gene segments that are located separately in the human genome. The HC gene results from the recombination of four different gene segments, the variable (IGHV), joining (IGHJ), diversity (IGHD), and constant (IGHC) gene segments, of which the first three encode the protein V_H_ domain. On the other hand, the LC gene is assembled by recombination of three gene segments, the variable (IGV_L_), joining (IGJ_L_), and constant (IGC_L_) gene segments, with the first two encoding the protein V_L_ domain (**Figure 2**). The immunoglobulin HC locus (IGH) is located in the long (q) arm of chromosome 14, regions 32.33 (14q32.33), while the immunoglobulin κ (IGK) and Λ (IGL) loci are located in the short (p) arm of chromosome 2 (2p11.2) and long (q) arm of chromosome 22 (22q11.2), respectively. The number of functional gene segments comprised at the IGH, IGK, and IGL loci varies across haplotypes.

The IGH locus spans ∼1.25 Mb and comprises 38-46 IGHV, 23 IGHJ, 6 IGHD, and 9 IGHC gene segments. The IGK locus spans ∼1.7 Mb and comprises 34-38 IGKV, 5 IGKJ, and only one IGKC gene segment. The IGL locus spans ∼1.1 Mb and comprises 29-33 IGLV, 4-5 IGLJ, and 4-5 IGLC gene segments (31) (http://www.imgt.org/IMGTrepertoire/; data accessed on March 8^th^, 2023). In the IGK locus, the IGKV gene segments are grouped into two clusters, termed distal (centromeric) and proximal (telomeric) clusters, separated by 800kb. In the telomeric direction, the proximal cluster is followed by the IGKJ cluster and them the only IGKC gene segment (31). The structure of the IGL locus is a little different since the IGLV gene segments are grouped into three clusters, termed C, B, and A (from centromeric to telomeric direction), which are followed by the IGLJ-IGLC cluster that comprises a polymorphic number of highly similar IGLJ-IGLC pairs (31) (**Figure 2**). Based on the degree of sequence similarity, the IGKV and IGLV gene segments have been subclassified into seven κ (κ1 to κ7) and ten Λ (Λ1 to Λ10) V_L_ subgroups, respectively. These subgroups consist of phylogenetically closely related V_L_ gene segments that were generated in relatively recent events of gene duplication and divergence (32). The number of V_L_ gene segments per subgroup varies from only one, as for subgroups Λ6 and κ4, to twenty in the case of subgroup κ1 (31, 33).

## 3 Mechanism for generation of antibody diversity

The adaptive immune system of jawed vertebrates evolved to generate a highly diverse repertoire of antibody specificities, which is achieved by two main mechanisms: 1) V(D)J genes combinatorial recombination to generate functional HC and LC genes, and the subsequent combination of the repertoire of HCs and LCs in the repertoire of B lymphocytes, and 2) SHM. V(D)J gene recombination is an antigen-independent mechanism of antibody diversification. In contrast, SHM is an antigen-dependent mechanism since it occurs after the activation of the mature B-cell by the antigen and its subsequent migration to the germinal centers of secondary lymphoid organs. SHM primarily targets the CDRs of antibodies, leading to affinity maturation. The antibody repertoire is further expanded by the addition of non-templated (N) nucleotides into the junctions between the V-D and D-J in HC gene, and although less extensive, also in the V-J junction in LC gene (34), a reaction catalyzed by the terminal deoxynucleotidyl transferase (TdT) (35). Other secondary mechanisms of antibody diversification are the V(DD)J recombination, SHM-associated insertions and deletions, and affinity maturation and antigen contact by non-CDR regions of the antibody (36).

### 3.1 V(D)J gene recombination

The V(D)J genes recombination begins in pro-B lymphocytes and progresses in discrete steps linked to specific stages of the B cell ontogeny pathway (**Figure 2**). A key player in this process is a lymphoid-specific enzyme called Recombination-Activating Gene (RAG) recombinase, a Y-shaped heterotetrameric complex made by two distinct subunits RAG1 and RAG2 (37–39). RAG1 is a 1040 amino acid multidomain protein highly homologous to many eukaryotic transposases, or retroviral integrases. It determines most of the catalytic and DNA binding capacity of the RAG complex. On the other hand, RAG2 is 527 amino acids in length and has no homology to any known viral transposase or integrase. Its role in V(D)J gene recombination is not well understood, but the evidence indicates that it is required for RAG complex DNA binding and catalysis (40, 41). Other components of the V(D)J genes recombination machinery are ubiquitous in cells since many of them form part of the non-homologous end-joining (NHEJ) DNA damage repair pathway (42, 43).

V(D)J gene recombination is directed by recombination signal sequences (RSSs), located immediately on the 5’ and/or 3’ flanks of the V, D, and J genes segments. An RSS comprises a highly conserved heptamer (consensus sequence 5’-CACAGTG-3’) and a conserved nonamer (consensus sequence 5’-ACAAAAACC-3’) separated by a poorly conserved spacer sequence of 12 or 23 nucleotides. This architecture determines two types of RSS, 12- and 23-RSS. The RSS located on the 3’ and 5’ flank of the IGHV and IGHJ gene segments, respectively, are of the 23-RSS type, while the IGHD gene segments bear a 12-RSS at both the 5’ and 3’ ends. The V(D)J gene recombination machinery strongly favors the recombination of gene segments with RSS of a different type, which is termed the 12-23 rule. This rule governs the V(D)J gene recombination fidelity, determining that an IGHV cannot directly recombine with an IGHJ gene segment, while an IGHD gene segment can recombine with either an IGHV or an IGHD. However, violation of the 12/23 rule occurs in a fraction of B lymphocytes under physiological conditions, which is believed to contribute to the diversification of the antibody repertoire (36).

RAG recombinase initiates V(D)J gene segments recombination by binding to either a 12-RSS or a 23-RSS and introducing a double-strand break (DSB) in the boundary between the RSS and the coding region of the target gene segment. The generation of a DSB is the signal that activates a group of proteins collectively called the DNA damage response. There is evidence that the High Mobility Group Box 1 (HMGB1) protein plays a critical role in this step, by binding to the RAG-DNA complex (44) and stabilizing a highly bent RSS conformation during catalysis (45, 46). Then, RAG-HMGB1 complex scans the locus and captures an RSS of a different type to form a paired complex, followed by nicking of the DNA chain (39). According to the 12/23 rule, if the RSS bound by the RAG-HMGB1 complex is a 12-RSS, then the RSS that will be captured in this step, and with which the recombination reaction will proceed, is a 23-RSS or vice versa. The DNA nicking generates a free 3’-hydroyl that attacks the opposite strand by transesterification, a reaction that requires Mg^2+^, and results in a covalently sealed hairpin at gene segment ends and the cleaved RSS ends. Factors in the NHEJ DNA repair pathway, such as the KU70/KU80 heterodimer, DNA PKcs, endonuclease Artemis, DNA polymerases µ and Λ, DNA ligases IV, X-ray cross-complementing Group 4 (XRCC4), TdT, and XRCC4-like factor (XLF) are then recruited to the coding ends. In the first step, the sealed hairpins are opened by the endonuclease activity of the Artemis : DNA-PKcs complex (47, 48), and then, the recombining gene segments are joined, forming imprecise coding joint that contains added nucleotide. The signal ends are also ligated to form a signal joint (42, 43). Most of the previously mentioned factors belong to the canonical NHEJ DNA repair pathway; however, DNA double-break repair can proceed by an alternative NHEJ pathway that involves a different set of factors (42, 43). V(D)J gene recombination results in gene segment joining and the deletion or inversion without deletion of the intervening DNA. Whether the V(D)J gene recombination occurs by a deletional, or inversional mechanism depends on the relative orientation of the two RSS driving the process (43).

Once V(D)J gene recombination has been successfully accomplished, the immature naïve B lymphocytes transcribe the HC and LC genes and express the encoded immunoglobulin as a membrane receptor (**Figure 2**). Initially, the HC variable region exon is transcribed in association with the immediately downstream Cμ exons, and in some cells, Cδ exons. This is accomplished by alternative splicing of the HC gene transcript, which comprises both µ and δ IGHC exons fused to IGHJ exon (43, 49).

V(D)J gene recombination is linked to specific differentiation stages of B and T cell ontogeny. Mutations that completely suppress the functions of RAG1/2 proteins cause severe combined immune deficiency, due to the developmental arrest at the progenitor stages of both T and B lymphocytes (50–54). In addition, it is important to highlight that V(D)J gene recombination proceeds *via* DSBs, one of the most toxic DNA lesions, and the rearrangement of relatively large DNA segments. These molecular events imply a latent risk of genome instability and the development of malignant diseases, such as leukemias and lymphomas, if they occur uncontrolled. (55–61). Thus, V(D)J gene recombination is tightly regulated by several mechanisms operating at various levels, to ensure that it occurs at the appropriate cell lineage and stage of development. Regulation is achieved by modulating the expression and activity of the key proteins RAG1/2, as well as through chromatin remodeling and control of the accessibility of the immunoglobulin gene segment locus to the recombination machinery (62).

Chromatin remodeling catalyzed by specific cellular factors turns physically accessible clusters of enhancers and promoter regions of *rag1* and *rag2* to the cooperative binding of transcription regulators and mediators, such as Pax5, Ets1, Ikaros, and Irf4 for B cells, and Bcl11b, Tcf1, Runx1, Ikaros, and Gata3, for T cells. This results in complex structures known as super-enhancers (SEs), that efficiently promote the transcription of *rag1* and *rag2*, in a lineage- and stage-specific manner (62).

### 3.2 Somatic hypermutation and class switch recombination

The actual size of the human antibody repertoire remains a matter of debate. Theoretical combinatorial calculations estimate it in the range of 10^12^ to 10^18^ different clones (63–65). However, there are several factors that limit the actual size of the antibody repertoire, such as the total number of B cells in a single human body, which has been calculated in 10^11^ (66). Furthermore, not all Ig gene segments rearrange with the same frequency and, at the protein level, not all HC and LC chains can efficiently pair to form a stable and functional antibody molecule (65, 67). More conservatively, it is estimated that the V(D)J gene segment recombination generates a naive repertoire of the order of 10^5^-10^6^ different antibodies, consisting mainly of low-affinity IgM, a fraction of which are polyreactive (68, 69). This repertoire is further diversified to in excess of 10^9^ different antibodies by two main mechanisms, SHM and antibody class switch recombination (CSR) (70). SHM generates multiple single nucleotide substitutions in and around the productively rearranged V(D)J gene segment, resulting in antibodies with higher antigen binding affinity but the same specificity (13, 43, 70). In contrast to V(D)J gene recombination that occurs in the omentum and fetal liver during B cell development, and in bone marrow in adult individuals, SHM occurs in the germinal centers of the secondary lymphoid organs. There, an iterative alternation of SHM and antigen-mediated selection leads to antibody affinity maturation (13, 43, 70).

The key enzyme in SHM, as well as in CSR, is the activation-induced cytidine deaminase (AID), a 198-amino-acid protein member of the AID/apolipoprotein B mRNA-editing enzyme-catalytic (APOBEC) family (71). AID is a single-strand DNA-specific deaminase that catalyze the conversion of DNA cytidines (dC) to uridines (dUs), with no observable activity on double-strand DNA, RNA, or RNA : DNA hybrids. This enzyme is expressed at high levels in germinal center activated B cells with a very strict temporal and spatial regulation (71). This determines that, in normal conditions, SHM of HC and LC genes occurs during only a narrow window of B cell development (70, 72, 73). SHM occurs through a two-step process. In the first step, AID catalyzes the hydrolytic deamination of dC in single-stranded DNA into dU and ammonia, introducing single nucleotide substitutions in a stepwise manner at a frequency of around 10^−3^ per base pair per generation (43, 70, 73). This is a million times higher than the normal mutation rates in non-Ig genes (43, 70, 74). AID preferentially targets dC located at hot spot motifs such as WRCY (when W = A or T, R = A or G, Y = C or T), with AGCT being a preferred motif (75). In opposition, the cold spot SYC (S = G/C; Y = C/T) is avoided, being mutated at frequencies 2 to 10-fold lower than the hot spots (70, 75). Codons prone to amino acid change tend to be concentrated in CDRs, as compared to FRs, which are thought to be an evolutionary solution to meet competing demands for diversification and preservation of antibody integrity. However, differences between κ and Λ LCs have been observed. It was found that in germline Λ LCs, codons at the CDRs are prone to replacement mutations, whereas, in the FR, the opposite is true, like in HC. In contrast, in germline κ LCs, codons in both CDR and FR are more prone to replacement mutations (76). It was hypothesized that the observed differences between κ and Λ light chains represent different evolutive strategies to balance diversity and stability in an immune response (76). On the other hand, as we will see later, these differences may also determine different pathogenic behavior of κ and Λ LCs in circumstances in which the LC is secreted in a free state (77, 78).

Generation of dU by AID-dependent deamination of dC results in U:G mismatch. The type of change that results from AID-dependent dC deamination depends on which DNA repair pathway processes the U:G lesion; the mismatch repair (MMR) or the base excision repair (BER) pathway (72). Notably, in SHM, error-prone pathways are favored instead of the canonical pathways that catalyze high-fidelity U:G repair (72). In the process dependent on the MMR pathway, U:G mismatch is detected by MutSα (MSH2/MSH6 heterodimer) (79), which recruits apurinic/apyrimidinic endonuclease 2 (APE2) and exonuclease 1 (Exo1). APE2 then cleaves the DNA 5’ of the mismatch, creating a DNA nick that serves as a point of entry for the Exo1, which initiates resection from the nick going past the mismatch site and creates an extended patch of ssDNA (43, 70, 80). Events in the multi-molecular complex formed at the site of DNA lesion result in the substitution of the high-fidelity DNA pol δ and pol ϵ by error-prone translesion DNA synthesis (TLS) polymerases, such as pol θ, polη, Rev1, and pol ζ (81). Polη is thought to play a major role in this step (73). This factor is recruited through proliferating cell nuclear antigen (PCNA) ubiquitination to resynthesize the DNA gap and introduce mutations, which are mainly at A:T pairs. Polymerase η is a mutator of A/T and C/G pairs *in vitro*, with one-quarter of its errors being introduced at C/G pairs (73, 82). The average base substitution error rate of human polymerase η is ∼3.5 × 10^−2^ (82), orders of magnitude higher than those of replicative DNA polymerases, which are in the range from 10^−6^ to 10^−4^. After the gap is closed by DNA polymerases, ligase 1 finalizes repair by sealing the break.

When an U:G mismatch is repaired by an error-prone BER pathway, dU is recognized by uracil-DNA glycosylase (hUNG), which removes dU from DNA (83), leaving an abasic site (AP site) that is recognized by APE1/2 (84). APE1 and/or APE2 are believed to generate the strand break by incising the AP site generated by hUNG. Interestingly, a recent study demonstrated that hUNG can catalyze DNA backbone cleavage after uracil excision, which suggests that the first two steps in uracil BER can be performed by this factor (83). In a canonical BER, PARP1 is activated, and scaffold protein XRCC1, Polβ, and DNA ligase IIIα are recruited (85). Polβ then removes the 5’ deoxyribose and inserts a single nucleotide, followed by ligation to complete the repair (84). In non-canonical, error prone BER during SHM, some abasic sites serve as a non-informative template (84). REV, a Y-family DNA polymerase recruited by PCNA ubiquitination as well, inserts dCMP into the new DNA strand opposite the abasic site. After a further round of DNA replication, this can result in a stable transversion mutation at the site of the original C:G base pair (43, 70, 84).

On the other hand, CSR allows the generation of antibody isotypes with different effector functions, which contributes to the effectiveness of the humoral response (86). CSR is a DNA deletional-recombination that removes the µ IGHC exons and places the functional VDJ segment into proximity with the exons of downstream HC constant regions (43, 80). This causes the replacement of membrane-bound IgM and IgD by membrane-bound IgG, IgA, or IgE; better protective antibodies. Such change in antibody class is accomplished without altering the V_H_(D)J_H_ or V_L_J_L_ exon assemblies for HC and LC, respectively, which is the base of the one-cell-one-antibody paradigm (87). CSR is driven by switch (S) regions, G-rich repetitive DNA elements located upstream of each CH gene segment, except the CHδ (88). Like SHM, CSR proceeds through AID-introduced DSBs, but in contrast to SHM that mostly targets the V_L_ region, CSR is restricted to the CH locus. As mentioned previously, AID is a ssDNA-specific deaminase, therefore, transcription is essential for the AID action. Each individual CH gene segment, except CHδ, are organized as transcription units, consisting of a cytokine/activation-inducible promoter, followed in downstream direction by an intermediate (I) exon, an S region, and CH exons. Activation of transcription through an individual CH gene segment by cytokine treatment induces CSR to that isotype (89, 90). On the other hand, expression of germline CH µ gene segment transcripts is constitutive and unaffected by mitogen or cytokine treatment (91). At the onset of CSR, the cytokine/activation-inducible promoter of the target CH gene segment is selectively activated, which results in a non-coding germline transcript that initiates at the I exon, proceeds through the S region and terminates downstream of the corresponding CH gene segments. This allows generation of RNA : DNA hybrid structures, such as R-loops, exposing stretches of ssDNA that serve as substrates for AID. There is evidence that AID features an intrinsic preference for G-quadruplex (G4)-containing DNA (92). G4-DNA structures formed by G-rich sequences present in S regions appear to induce CSR by promoting AID binding (93, 94). The binding of AID to the active S region requires the participation of 14-3-3 adaptor proteins (95) and also involves the recruitment of protein kinase A (PKA) (96). PKA phosphorylate AID at Ser38 (96), generating a binding site for RPA, which enhance the deamination activity of AID (97). AID converts dC exposed in the displaced G-rich nontemplate strand within the transcribed targeted S regions to dU, with a specific affinity for WRCY motifs (98). The AID-generated dU:dG mismatch can be processed *via* either BER or MMR pathways. However, the evidence support the notion that BER pathway is preferred in CSR, in particular when two dU:dG mismatches are closely spaced on opposite DNA strands (98–100). *Via* BER, DSBs are generated by subsequent dU deglycosylation by UNG that creates an abasic site. Then, the abasic site is recognized by the apurinic/apyrimidic endonuclease APE1, generating a nick. The evidence indicates that MMR pathway plays a less relevant role in CSR than BER (98). It is believed that MMR is involved in the conversion of single-strand breaks (SSBs) on opposite DNA strands that are located distal each other into DSBs (98). It has been suggested that Msh2–Msh6 may bind to dU:dG mismatches that have not been processed by UNG and recruit EXO1 to excise DNA from the nearest 5′-SSB to the mismatch, creating a DSB with a 5′-overhang (98, 101).

End-joining of DSBs between donor and acceptor S regions is carried out mostly by the C-NHEJ DNA DSB repair pathway, which employs the Ku70/80 complex (Ku) for DSB recognition and the XRCC4/DNA ligase 4 (Lig4) complex for ligation. However, A-NHEJ DNA repair pathway is believed to also participate in this step under certain circumstances. The result is the deletion of the expressed IgM/IgD CH gene segments, which are replaced by a new CH gene directly downstream of the VDJ(H) exon.

As for V(D)J gene recombination, B cells use mechanisms operating at different levels to keep both SHM and CSR under strict regulation. At the cell level, SHM is induced by specific stimuli such as crosslinking of the BCR, the engagement of CD40 and co-engagement of CD80 and CD86 on the B-cell surface by CD154 and CD28 expressed on the surface of activated T cells, and cytokines, such as interleukin 4. These stimuli specifically upregulate AID by means of, among others, epigenetic mechanisms mediated by histone-modifying enzymes acting at the promoter regions of AID (43). AID gene is the target of the gene regulatory network composed of several transcription regulators and orchestrated by BACH2 and IRF4 that promotes SHM and CSR of antibodies and simultaneously represses plasma-cell differentiation (102–104). Epigenetic mechanisms induced in response to B cell activating stimulus also activate miRNAs like mir-16, mir-155, and mir-181b that decrease the expression of AID by binding to and degrading complementary sequences of the mRNA (105–107). As mentioned before, AID is also regulated by phosphorylation in Ser38 by cAMP-dependent protein kinase (PKA) that increases AID activity at the Ig HC switch regions (43, 108). The activity of AID can be also regulated by phosphorylation at threonine 140 (Thr140) by protein kinase C (PKC) family members, a mechanism preferentially affecting SHM (109). Serine 3 (Ser3) is another phosphorylation site that causes a reduced activity of AID, an effect that can be reversed by dephosphorylation catalyzed by protein phosphatase 2A (PP2A) (110). AID is also regulated by a complex mechanism that controls the enzyme subcellular localization (111) and stability (112). AID is also cell cycle regulated by processes that involve checkpoint kinase 1 (Chk1) and 2 (Chk2), two factors that regulate both SHM and CSR but with opposite effects (113).

Regardless of the numerous overlapping regulatory mechanisms that control AID, the mutagenic activity of this enzyme can be inappropriately activated by several factors, such as chronic inflammation (114–116), hepatitis C virus infection (117, 118), and estrogen (119, 120). Aberrant activation of AID has been linked to the pathogenesis of autoimmune disorders (121) and several types of cancer (122), including colon cancer (114, 123, 124), hepatocellular carcinoma (125, 126), skin cancer (127, 128), oral squamous cell carcinoma (129), and multiple myeloma and other B cell malignancies (130–133). There is evidence that AID plays a role in the occurrence of chromosomal aberrations in monoclonal gammopathies, such as the translocation of chromosomes 11 and 14 [t(11;14)(q13;32)] (134). This chromosomal alteration is detected in 16%-24% of patients with multiple myeloma (135), a frequency that reaches ∼50%-60% in those with AL amyloidosis, constituting a distinctive characteristic of this disease (136–138). In AL amyloidosis, [t(11;14)(q13;32)] has a significant impact on therapeutic response and overall survival (139–141). AL amyloidosis with [t(11;14)(q13;32)] is associated with low plasma cell count but high serum-free LC level, which may influence the tissue distribution and burden of AL deposits (138).

## 4 Diseases caused by immunoglobulin light chains misfolding and aggregation

**Figure 3:**
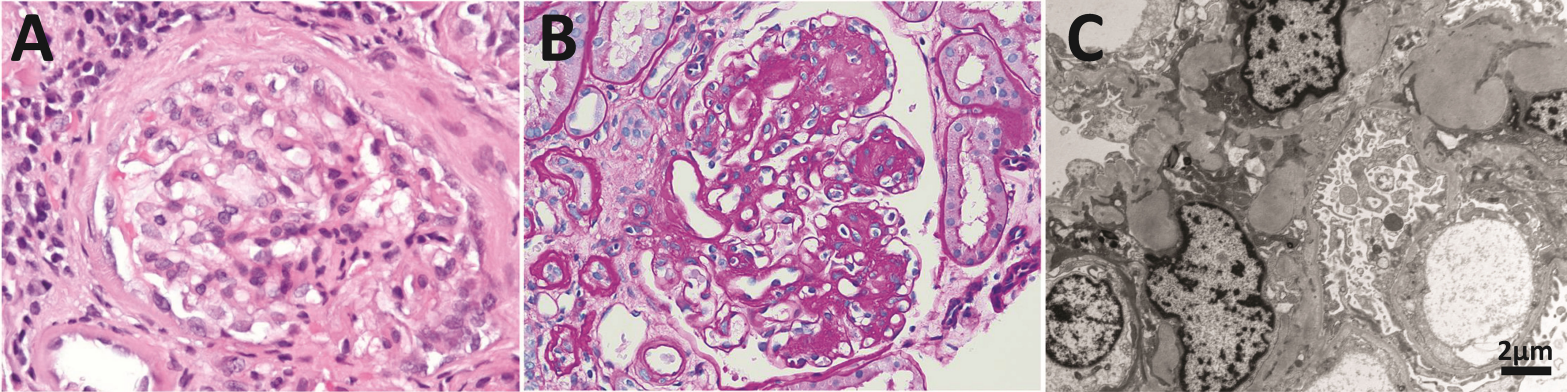
Anatomopathological features of light chain deposition disease (LCDD). **(A)** Hematoxylin & eosin (H&E) and **(B)** PAS staining of kidney biopsies from two patients in the early and late stages of the disease, respectively. Note in B the nodular mesangium with a markedly increased tenascin-rich extracellular matrix. **(C)** Electron microscopy (EM) analysis that shows expanded nodular mesangium with an increased extracellular matrix and punctate, powdery, ground-pepper-like extracellular deposits.

**Figure 4:**
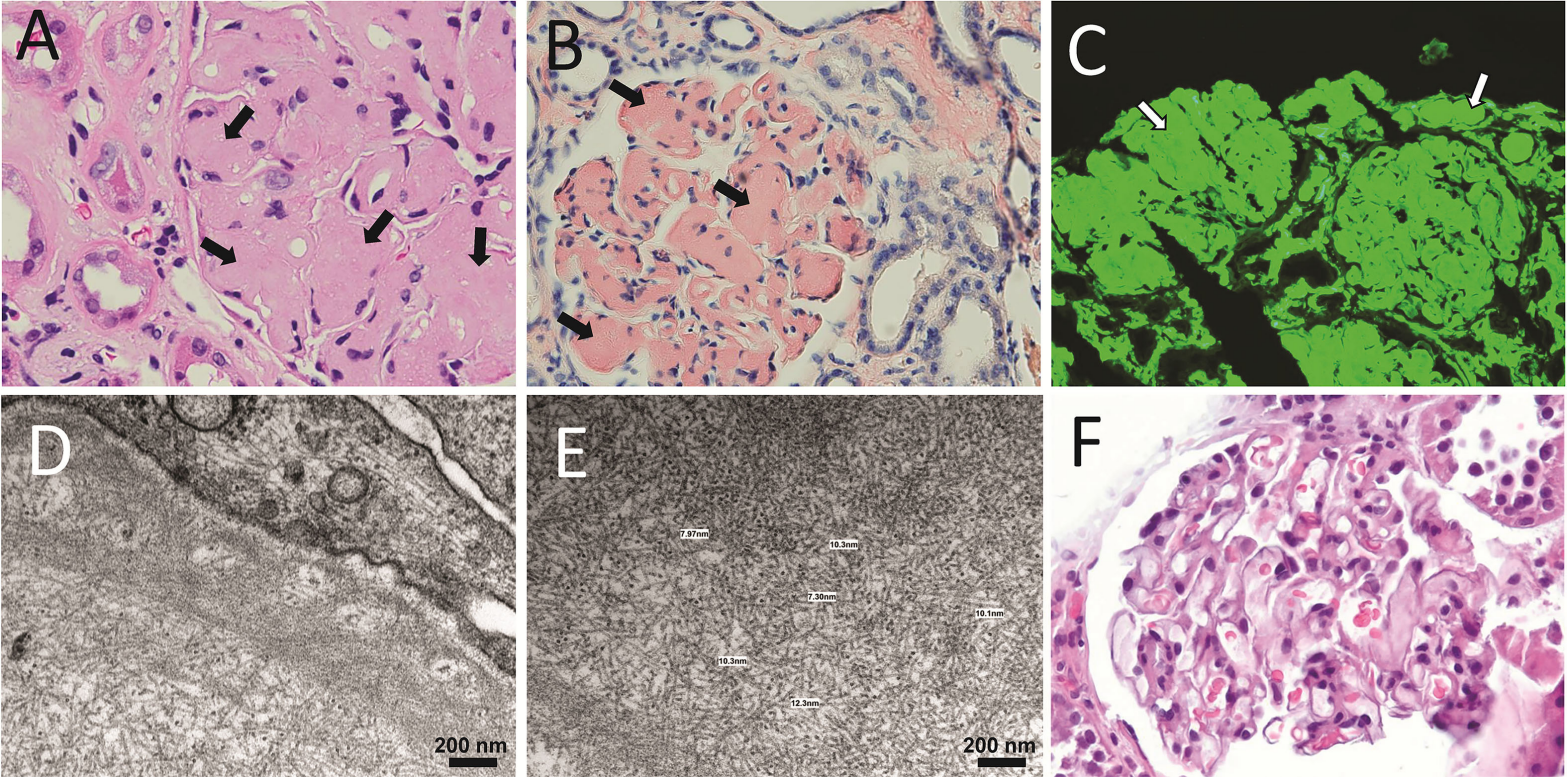
Anatomopathological characteristics of renal AL amyloidosis **(A–C)** H&E, Congo red, and immunofluorescence analysis for human κ LC of a renal biopsy of a κ AL amyloidosis patient in advance stage of the disease. **(D, E)** show images taken in EM analysis of the same sample shown in **(A–C)**. **(F)** The H&E staining of the renal biopsy of a Λ AL amyloidosis in early stage of the disease is presented for comparative purpose. In A, arrows indicate AL amyloid deposits stained with H&E that appear as a waxy, homogenous, eosinophilic material that has almost completely replaced the mesangium. In B and C, arrows indicate the same material stained with Congo red, and detected by the anti-human κ LC antibody, respectively. In C, the primary, and secondary antibodies were goat anti-human κ LC antibody and fluorescein-conjugated rabbit anti-goat IgG, respectively. Note in D and E the abundant randomly disposed fibrils with diameter in the range of 7.3 nm to 12.3 nm, characteristic of the AL amyloid fibrils.

In healthy individuals, most LCs are secreted as part of the antibody (**Figure 1**), the context in which the LC evolved to carry out its recognition function. The quaternary structure of antibodies represents a protective context for the LC against intrinsic and extrinsic deleterious factors that can promote misfolding and aggregation. The contacts at the HC/LC interface contribute to the LC thermodynamic stability, counterbalancing the destabilizing effect of somatic mutations (142–144). Also, the multidomain structure of the antibody prevents the LC from participating in edge-to-edge intermolecular H-bonding, reducing the risk of aggregation (145, 146). However, in patients with monoclonal gammopathies, the abnormally proliferating plasma cell clone overproduces the LC and secretes it in a free state into the bloodstream (147) (**Figure 2A**). In such a condition, the monoclonal LC, deprived of its natural protective context, is more susceptible to misfolding and aggregation (77, 78). LCs can form various pathologic aggregates that differ from each other in ultrastructural morphology, internal structure, and biological properties. Some LCs form crystals in the cytoplasm of the cells of the proximal tubules and disturb their absorptive capabilities, causing a disorder known as Fanconi’s syndrome (FS) (148). Other LCs reach distal nephrons, where they co-aggregate with the Tamm-Horsfall protein to form intratubular casts, a disorder termed myeloma (cast) nephropathy that is characterized by renal failure primarily by an obstructive mechanism (149). There is another group of pathogenic LCs that form non-ordered, amorphous aggregates that can deposit in any organ, although affect predominantly the kidneys, causing a disorder known as LC deposition disease (LCDD) (**Figure 3**). Finally, there is a group of LCs that displays a propensity to deposit in the extracellular spaces of organs and systems in the form of amyloid fibrils, whose distinctive characteristic is a structural core riches in β structure (**Figures 2A** and **4**). This causes LC-derived (AL) amyloidosis, the systemic form of amyloidosis most frequently diagnosed in Western countries (150, 151). As a systemic disorder, AL amyloidosis usually affects various organs, although the heart and kidneys are the most frequently involved.

The diversity of pathological aggregates that LCs form is a direct consequence of their sequence diversity and reflects the wide spectrum of conformation that these immunoglobulins can adopt in a context-dependent manner (143, 152–154). In this regard, AL amyloidosis, by far the most clinically heterogeneous of all systemic amyloidoses, is a good example of how LC sequence diversity translates into a wide spectrum of clinical courses (155, 156).

It is important to mention that not all monoclonal LCs aggregate *in vivo*. Several long-term follow-up studies of patients with Bence Jones idiopathic proteinuria have shown that not all develop AL amyloidosis, or any other disease associated with LC deposition (157–162). This indicates that the secretion of the LC in a free state, although a required condition, is not sufficient for LC aggregation to occur. It is the interaction between the structural and biophysical properties of the LC, both a function of its sequence, and factors of the tissue microenvironment that determine if and how LC aggregation occurs (77, 78, 163–166).

### 4.1 Genetic and structural factors that drive LC aggregation in human diseases

The intrinsic propensity of a monoclonal LC for pathological aggregation is determined by both the encoding V_L_ gene segment (167–171), and the somatic mutations (54, 152, 164, 172–176). The encoding IGV_L_ gene segment may confer to the LC the propensity to aggregate in a specific form, as well as in a specific organ or tissue (organ tropism). As far as we know, the first report of the association of a V_L_ subgroup with a monoclonal immunoglobulin deposition disease was done by Solomon et al. (177), who observed an overrepresentation of the Λ6 LCs in AL amyloidosis (177). Subsequent studies corroborated Solomon and colleagues’ findings and, furthermore, revealed the bias in the IGV_L_ gene segments usage that characterizes the AL amyloidosis (167, 168, 170, 178–180). Such bias is clearly observed in the collection of AL LCs sequences compiled in the AL-Base (http://albase.bumc.bu.edu/aldb). Currently, this curated database contains the sequence of 570 amyloidogenic LCs, ∼61% of which derive from only five IGLV gene segments (*IGLV1-44*, *IGLV2-14*, *IGLV3-1, IGLV6-57*, and *IGKV1-33*), These gene segments, in conjunction, account for only ∼7% of the human repertoire of κ and Λ IGV_L_ genes segments, composed by ∼70 elements (31). *IGLV6-57*, the only member of the Λ6 subgroup, is the most frequently used gene segment in the set of AL-associated LCs compiled in the AL-Base, since it accounts for 17.8% of the whole set (129 κ and 441 Λ) and 23% of those of Λ type (https://wwwapp.bumc.bu.edu/BEDAC_ALBase). Kourelis et al. established the use of immunoglobulin gene segments in a series of 701 and 120 patients with systemic and localized AL amyloidosis, respectively, the largest study to date on this topic (170). They found that, overall, the Λ gene segments *IGLV6-57*, *IGLV2-14*, *IGLV3-1*, and the members of the V_L_ gene segment subgroup κ1, which includes *IGKV1-33*, constituted 49% of the clones identified in systemic AL amyloidosis. Again, *IGLV6-57* was the most frequently used IGV_L_ gene segment, accounting for ∼23% of the systemic AL amyloidosis caused by a Λ LC (170). The strong association of *IGLV6-57* gene segment with amyloidosis is supported by two findings. One is the difference between its low frequency of expression (∼2%) in the normal repertoire of polyclonal bone marrow plasma cells expressing a Λ LC (168), and its ten-time higher frequency in AL amyloidosis (170). The second finding is the contrasting difference in the number of amyloidogenic and biopsy-proven non-amyloidogenic monoclonal Λ6 LCs reported in the literature (181). Currently, the AL-Base contains the sequence of 102 Λ6 amyloidogenic LC (Data retrieved on May 18^th^, 2023). For many years, JTO was the only monoclonal Λ6 LC for which clinical data excluding amyloid deposition was available. This protein was identified in the 90s in a patient with myeloma (cast) nephropathy without clinical evidence of amyloid deposition (182, 183). Nig-48 (Accession number P01722) is another monoclonal Λ6 LC classified in the AL-Base as derived from plasma cell dyscrasias (PCD) other than AL amyloidosis. However, in the original publication that reports this protein, no clinical data of the patient is informed. Therefore, it is unknown whether this protein caused AL amyloidosis (184). The near absence of biopsy-proven non-amyloidogenic monoclonal Λ6 LCs led to suggest a near absolute link between the Λ6 isotype and AL amyloidosis (169, 181). However, very recently, Nau et al., identified 7 monoclonal Λ6 LCs in patients with multiple myeloma without the diagnosis of AL amyloidosis (185). This study was aimed at validating a computational approach using the MiXCR suite of tools to extract complete rearranged IGV_L_-IGJ_L_ sequences from untargeted RNA sequencing data. This approach was successfully applied to whole-transcriptome RNA sequencing data from 766 newly diagnosed patients in the Multiple Myeloma (MM) Research Foundation CoMMpass study (185). While the study of Nau et al. suggests that PCD patients with a circulating monoclonal Λ6 LCs without amyloidosis could be more frequent that previously assumed, we think that it is important consider some facts in analyzing this report (185). Remarkably, the authors reported that only 14 (2%) of 705 patients were reported with the diagnosis of AL amyloidosis, a figure that contrasts with the incidence of secondary amyloidosis in MM patients reported in other studies, which varies from 25% to ∼40% (186–188). In addition, some of the 7 MM patients with a monoclonal LC Λ6 without a diagnosis of AL amyloidosis had signs and symptoms of renal failure and peripheral neuropathy, conditions frequently associated with AL amyloidosis (189). As the authors of this article state, it will be required Congo red staining and immunohistochemistry analysis with LC-specific antibodies in subcutaneous biopsy to rule out the diagnosis of AL amyloidosis in these patients (185).

On the other hand, several studies have consistently found that renal involvement is more frequent in AL patients with an *IGLV6-57*-derived LC, when compared with patients without (167, 170, 180). Trends suggestive of organ tropism have also been observed in other IGV_L_ gene segments (167, 170, 180).

Although several hypotheses have been proposed, no study has provided straightforward evidence of mechanisms or factors explaining the association of a few IGV_L_ gene segments with AL amyloidosis. In the case of *IGLV6-57*, structural factors encoded by the germline of this gene seem to play a role, since the rV_L_ protein with the germline sequence of *IGLV6-57* was shown to be intrinsically prone to aggregate as amyloid-like fibrils *in vitro* (169, 181). Indirect evidence obtained in a recent study suggests that the germline *IGLV3-1* gene also encodes a V_L_ protein highly prone to aggregate (190). On the other hand, evidence indicates that the association of *IGLV1-44* and *IGKV1-33* with amyloidosis cannot be explained solely by the properties of the germline V_L_ protein. Other not yet identified factors may be more relevant for these gene segments (181).

The association of certain IGV_L_ gene segments with AL amyloidosis may also reflect the preferential rearrangement of these genes in certain B lymphocyte populations driven by local factors (16, 167). Chronic antigenic challenge, either by foreign or self-antigens, along with local factors, may play a role in promoting the selection and expansion of B lymphocytes that preferentially rearrange these IGV_L_ gene segments (191). However, to our knowledge, there are no reports of antigenic specificity for a plasma cell clone implicated in AL amyloidosis.

Understanding what determines the association of some IGV_L_ genes with amyloidosis deserves further investigation, as mounting evidence indicates that the V_L_ gene segment encoding the amyloidogenic LC influences both the clinical presentation and outcome of patients with AL amyloidosis (167, 170, 179, 180).

Little is known regarding the IGV_L_ gene segment usage in other diseases caused by LC deposition, mainly because of the few numbers of LC sequences reported. The data suggest that the repertoire of IGV_L_ genes in LCDD and Fanconi syndrome is also biased to a few elements. Most of the LCs involved in LCDD are encoded by a restricted set of κ IGV_L_ genes that belong to κ1 (*IGKV1-5*), κ3 (*IGKV3-11* and *IGKV3-15)* or κ4 (*IGKV4-1*) subgroups (15, 192). Only two Λ LCDD LCs have been reported so far (193). Most of the LC involved in Fanconi syndrome identified so far were encoded by *IGKV1D-33/IGKV1-33* (O8/O18) or *IGKV1D-39/IGKV1-39* (O2/O12), two pairs of duplicated gene segments that belong to the κ1 subgroup (194, 195). LCs encoded by the κ3 gene segments *IGKV3-11* (L6), IGKV*3-15* (L2), and *IGKV3-20* (A27) have been reported in Fanconi syndrome patients too. We can conclude that at least part of the structural factors that drive LCs to form pathological aggregates are encoded in the germline IGV_L_ gene segment.

Interestingly, a highly restricted repertoire of IGV_L_ gene segments has been also reported in the monoclonal component present in patients with polyneuropathy, organomegaly, endocrinopathy, monoclonal gammopathy, and skin changes (POEMS) syndrome. In about 80% of the case, the circulating monoclonal LC is encoded by the Λ1 gene segments *IGLV1-40* and *IGLV1-44*. Furthermore, in almost all cases, the IGJ_L_ gene segment has been IGLJ3*02 (196).

### 4.2 Role of somatic mutations in light chain amyloid aggregation, cytotoxicity, and organ tropism

Early studies found that the amyloidogenic LCs tend to be thermodynamically less stable than their non-pathogenic counterparts (143, 152, 153, 172, 173, 183, 197–200). It has been consistently found that destabilizing somatic mutations, as well as stringent conditions of temperature, pH, or concentration of chaotropic salts, such as urea and guanidinium hydrochloride (GdnHCl), accelerate the kinetic of LC fibrillogenesis (199, 201–204). Thus, thermodynamic stability was early identify as a key driver of LC amyloid aggregation (152, 172, 173, 183, 205–207). It was predicted that a less thermodynamically stable LC is more likely to adopt non-native conformations prone to self-assemble into amyloid. This model confers non-native intermediaries a key role in the mechanism of LC amyloid aggregation, a key concept for understanding AL amyloidosis as a protein misfolding disease (172, 204, 208–210).

The recognition of somatic mutations as main actuators in AL amyloidosis pathogenesis resulted from studies that demonstrated their ability to destabilize the LC and promote its fibrillar aggregation (152, 172, 173, 198).

**Figure 5:**
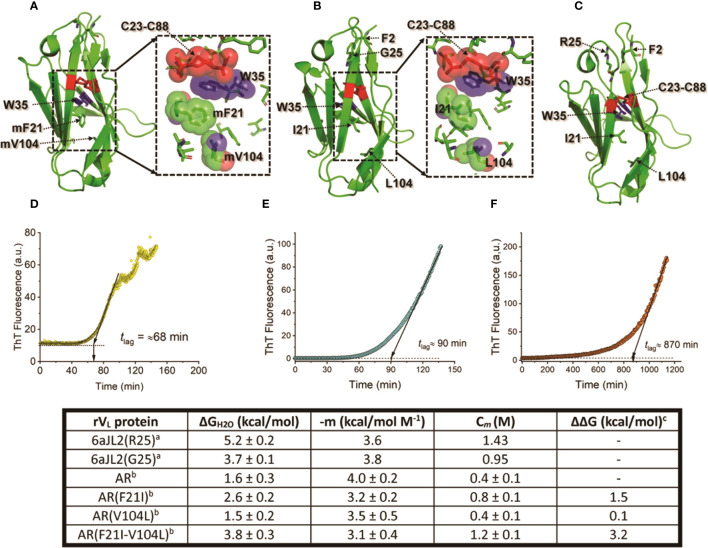
Impact of somatic mutations Ile21Phe and Leu104Val on the structure and biophysical properties of the amyloidogenic Λ6 LC AR. **(A)** to **(C)** show the crystallographic structure of the rV_L_ proteins AR, 6aJL2(G25), and 6aJ2(R25). Mutant residues (m) Phe21 and mVal104, and wild type Ile21 and Leu104 are represented in stick format and indicated. For reference purposes, the highly conserved Trp35, the disulfide bond Cys23-Cys88, Phe2, and the residue at position 25 are also represented in stick format and indicated. The inset in A and B show a more detailed representation of the spatial relationship of mPhe21, mVa104, Ile21, and Leu104 with non-polar residues that compose the core of the V_L_ domain fold. The mutant and wild-type residues, Trp35, and the disulfide bond Cys23-Cys88 are represented in a combination of semi-transparent spheres/sticks representation, to highlight the side chain-to-side chain interactions. Proteins 6aJL2(R25) and 6aJL2(G25) have the germline sequence of the *IGLV6-57* (Λ6) segments. They have the same sequence, except in position 25, which is an allotypic variation of the *IGLV6-57*. Variant G25 suppresses a cation-π interaction between Phe2 and Arg25, determining a less thermodynamically stable and more fibrillogenic protein (169). **(D-F)** show the *in vitro* fibrillogenesis of the rV_L_ Λ6 proteins AR, 6aJL2(G25), and 6aJL2(R2) performed at 200 µg/ml in presence of 20 µM thioflavin T. The protein solutions were incubated at 37°C with constant stirring of 350 r.p.m. with a 5x2 mm Teflon-coated magnetic micro-stir bar. Amyloid-like fibril formation was detected by serially measuring ThT fluorescence, exciting the sample at 450 nm, and recording the ThT fluorescence at 482 nm. The lag time (*t*_lag_) for nucleation was calculated by extrapolating the linear region of the hyperbolic phase back to the abscissa (213). The table in the bottom shows the thermodynamic parameters of the rV_L_ protein AR, its single and double mutants AR(F21I), AR(V104L), and AR(F21I-V104L), and the germline proteins 6aJL2(G25) and 6aJL2(R25) determined by reversible unfolding with increasing concentration of guanidinium hydrochloride (GdnHCl). **(A)** Data were taken from reference (205). **(B, C)** Data were taken from reference (211). ΔΔG values were calculated considering AR as the wild type; positive values indicate a higher stability than that of AR. The structures shown in **(A)** to **(C)** were prepared with PyMOL (PyMOL Molecular Graphics System, Version 2.5.2, Schrödinger, LLC) using PDB 5IR3 for rVL AR, PDB 5C9K for rVL 6aJL2 (G25), and PDB 2W0K for rVL 6aJL2 (R25).

These studies also showed that the impact of somatic mutations is context-depend. This means that the mechanism by which mutations promote LC aggregation varies depending on the residues that are involved and the structural features of the LC itself (152). Some mutations have been shown to promote LC amyloidogenesis by disrupting the hydrophobic core of the V_L_ domain, destabilizing the domain fold (211). The Λ6 LC AR, the first AL protein whose amino acid sequence was determined, illustrates well this mechanism (212). This protein showed to be highly unstable in both temperature and GdnHCl equilibrium unfolding experiments (205). The V_L_ protein AR also readily aggregated into amyloid-like fibrils at physiological-like conditions of temperature, pH, and ionic strength; with faster kinetics than that of its germline counterpart protein 6aJL2(G25) and the highly amyloidogenic Λ6 V_L_ Wil (205). Protein AR also elongated homologous seeds highly efficiently. According to the thermal unfolding curve, the unfolded fraction of the rV_L_ AR at 37°C was estimated at ∼80%, which can explain its high aggregation propensity (205). AR differs from its germline counterpart in fourteen positions. However, it was shown that the restoratives single- Phe21Ile and double-mutant Phe21Ile/Val104Leu, both located in the hydrophobic core, restored ∼50% and ∼100%, respectively, of the thermodynamic stability lost in the V_L_ AR relative to its germline counterpart (**Figure 5**). As predicted, both mutants were significantly less fibrillogenic than AR, displaying aggregation kinetics like that of the germline counterpart (211). This study showed how detrimental somatic mutations that disturb the structural core of the V_L_ domain can be. At the same time, it raises the question of the role of this type of mutation in the LC recognition function. It cannot be ruled out that mutations such as those identified in protein AR improve the recognition properties and/or biological function of the antibodies by increasing the conformational flexibility of the V_L_ (214–218).

Other mutations have been shown to promote aggregation by altering the homodimer interface that LCs tend to form in solution (**Figure 1E**), shifting the equilibrium towards free monomers, which are typically thermodynamically less stable (219–221). This finding is in line with studies showing that the association of the LC in a stable dimer protects it from misfolding and aggregation (222, 223). Hence, stabilizing the LC dimer by small molecules that bind at the dimer interface is one of the strategies being explored to inhibit LC amyloidogenesis (224, 225)

Somatic mutations can also promote amyloidogenesis by shifting the kinetics of unfolding/refolding of the LC, as well as altering the internal dynamic of the protein (175, 226). It is believed that this effect may favor the accumulation of non-native species that nucleate the fibrillogenesis. In a recent study, Oberti et al. compared the structural and physicochemical properties of thirteen sequence-diverse full-length LCs and founds that the ability to form amyloid *in vivo* correlated with both low thermodynamic stability and high protein dynamics (227). Remarkably, no significant correlation was observed between hydrophobicity, structural rearrangements, and the nature of the LC dimeric association interface with the propensity to form amyloid (227).

On the other hand, Martin-Argany et al. found that highly destabilizing mutations that shift the equilibrium toward the unfolded state can inhibit, rather than promote, the amyloidogenesis of κ1 LCs (207). This finding supports the notion that a partially folded intermediate, and not the totally unfolded state, is the most amyloidogenic species in the LC unfolding pathway. However, evidence suggests that this may not apply to all LCs. It was observed inverse correlation between the unfolded fraction at 37°C, as calculated from the thermal unfolded curve, and the latency time (*t*_lag_) of *in vitro* fibrillogenesis of a group of four Λ6 LCs, which included the highly unstable protein AR and its germline counterpart 6aJL2(G25) (205). As mentioned before, the unfolded fraction of AR at 37°C is ∼80%. This suggests that, at least for Λ6 LCs, highly disordered conformer(s) may be efficient in triggering aggregation *in vitro*, contrary to what seems to occur in κ1 LC. These differences probably reflect the different contributions of factors such as somatic mutations and structural properties encoded in the germline V_L_ domain, which differ greatly between κ and Λ LC subgroups. (77, 78, 181).

**Figure 6:**
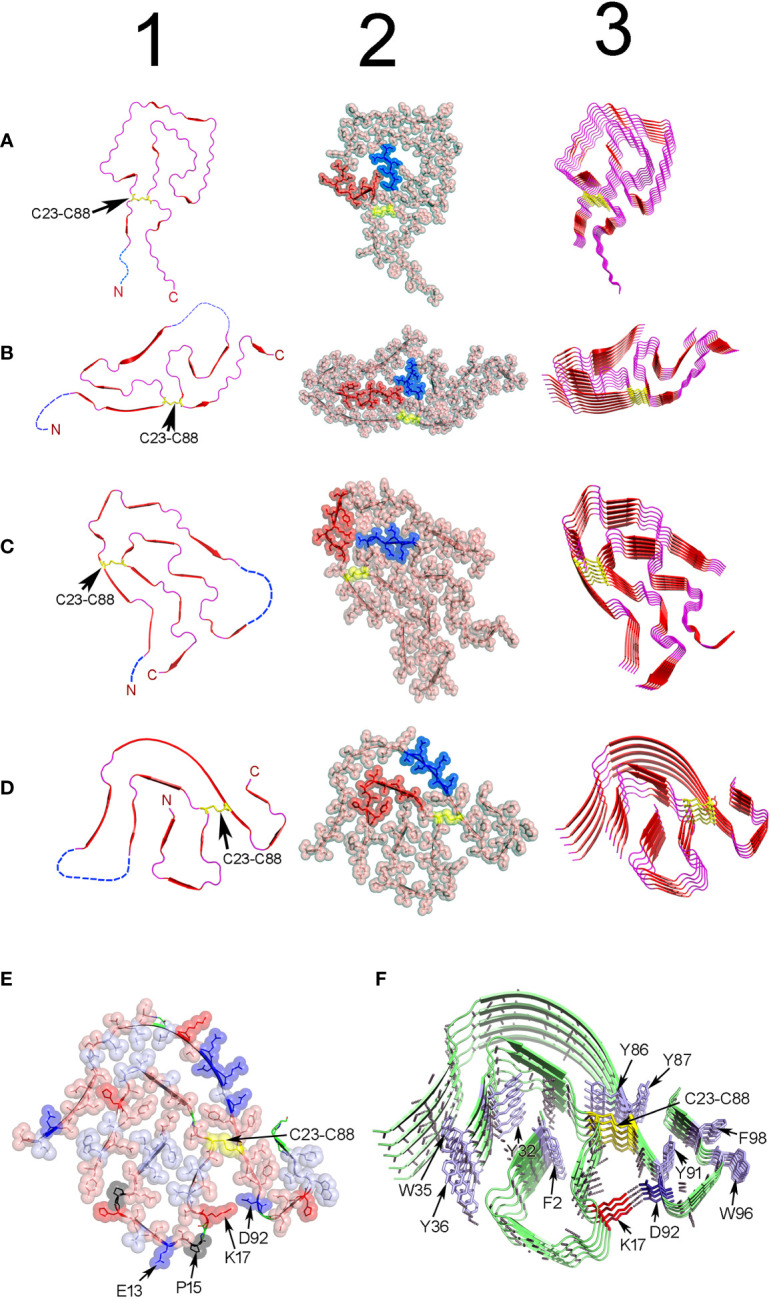
Structure of ex-vivo AL fibrils determined by cryo-EM. **(A)** AL fibril formed by a Λ1 LC encoded by the V_L_ gene segment *IGLV1-44*. PDB ID 6IC3 (233). **(B)** FOR001 AL fibril formed by a Λ1 LC encoded by the V_L_ gene segment IGLV1-51*02. PDB ID 7NSL (234). **(C)** FOR005 AL fibril formed by a Λ3 LC encoded by the V_L_ gene segment IGLV3-19*01. PDB OD 6Z1O (235). **(D)** AL fibril formed by the Λ6 LC AL55 encoded by the V_L_ gene segment IGLV6-57*02. PDB ID 6HUD (232). The four AL fibrils were extracted from cardiac deposits of patients with cardiac AL amyloidosis. Column 1 shows cartoon representations of the V_L_ domain polypeptide chain conformation in the amyloid fibrils. Regions in β-strand are shown as arrow and colored red. Disordered regions are represented as dashed lines in an arbitrary arrangement. Column 2 shows the same structures of column 1, but with the projection of the amino acid side chains in a combination of semi-transparent spheres/sticks representation to highlight the side chain-to-side chain interactions that contribute to fibril stability. The amino acid residues spanning the CDR1, and the loop connecting the β-strands E and F (loop E-F) are colored red and blue, respectively. Column 3 shows the assembly of the AL fibrils by stacking the monomers one on top of the other, with parallel in-register arrangement of β strands. In all structures, the conserved intradomain disulfide bond Cys23-Cys88 is represented in sticks or spheres format and colored yellow. N and C stand for N- and C-terminal. **(E)** and **(F)** show a cartoon representation of the same AL Λ6 fibril shown in D, with a detailed representation of the different types of interactions that stabilize the fibril structure. In **(E)**, the amino acid side chains are shown in a combined semi-transparent spheres/stick format, using the following color code: blue for negatively charged (acid) amino acids, red for positively charged (basic) amino acids, salmon for polar non-charged amino acids, light blue for non-polar amino acids, black for proline, and yellow for cysteines. Some residues are indicated for structural reference. Note that non-polar side chains tend to cluster buried in the fibril core, while charged and polar non-charged amino acids tend to locate in the solvent exposed surface. The image in **(F)** shows the stacking of the AL55 LC monomers, one on top of the other, with parallel in-register arrangement of β strands. The H bonds between the peptide backbones of adjacent monomers are shown as black dotted lines. Aromatic amino acids interacting by intermolecular stacking are shown. In both images, the conserved intradomain disulfide bond Cys23-Cys88 (colored yellow) and the fibril-specific salt bridge formed by Lys17 and Asp92 are shown. Note that this salt bridge is intermolecular, that is, it is established between residues located in monomers that are contiguous in the fibrillar structure. Regions in β-strands are represented as arrows. Amino acids are identified using a one-letter code. All structures were prepared with PyMOL (PyMOL Molecular Graphics System, Version 2.5.2, Schrödinger, LLC).

Recently, Peterle et al. published a study aimed at identifying the structural factors driving the high propensity of Λ6 LCs to deposit as amyloid (228). They found that the conservative mutation Asn32Thr destabilized the LC, an effect that propagated across the V_L_ domain increasing the dynamics of regions ∼30Å away from the substitution site. This effect caused the CDR1 to be less protected in hydrogen-deuterium exchange (HDX) mass spectrometry (MS) experiments, indicative of a more flexible loop. In fact, the CDR1 was the region that displayed the lowest protection in HDX-MS experiments in all Λ6 LCs studied by them (228). This is a relevant finding, considering that the CDR1 of the Λ6 LCs contains a highly pro-amyloidogenic hot spot that is predicted to play a key role in the aggregation mechanism (229). Pro-amyloidogenic hot spots are short segments in proteins that can drive amyloid aggregation in a sequence-dependent manner. It is believed that they play a key role in triggering the aggregation reaction, which is why they are considered potential therapeutic targets (230). Structural studies have shown that the CDR1 of Λ6 LCs undergoes extensive conformational adjustment in the amyloid state. This involves a helix-to-β-strand transition that allows the CDR1 to be placed in the inner core of AL fibrils (231, 232) (**Figure 6**). Therefore, it is plausible that mutations in the CDR1, or in residues that contribute to stabilizing the native fold of this loop, may trigger amyloid aggregation by promoting unprotected exposure of the CDR1 pro-aggregation hot spot to intermolecular contacts, as well as by facilitating the refolding of this loop into its amyloid conformation (78, 231, 232). Since a fibrillogenic hot spot spanning the CDR1 was shown to be also encoded by several κ and Λ germline IGV_L_ gene segments, we anticipate that the above-mentioned mechanism are also relevant to LCs other than Λ6 proteins (229).

Peterle et al. hypothesized that the observed long-range effects of the somatic mutation Asn32Thr may be relevant to the recognition function of the LC in the antibody (228). Somatic mutations that modulate the antibody conformational flexibility has been suggested to contribute to biologically relevant properties, such as cross-reactivity and broad neutralizing capacity against surface proteins of diverse strains of rapidly evolving viral pathogens, such as SARS-CoV 2 (218, 236, 237). Thus, the study conducted by Peterle et al. highlights the potentially deleterious consequences of somatic mutations which, playing a beneficial role in enhancing antibody recognition properties, can cause highly disabling diseases by promoting LC aggregation (217).

The evidence supports the idea that the V_L_ domain determines the potential of the LC to form amyloid. This put the C_L_ domain out of the focus of most research groups interested in the molecular pathogenesis of AL amyloidosis. However, it has been shown that the C_L_ modulates the unfolding/refolding kinetics of the LC, influencing the mechanism of aggregation (238, 239). Moreover, a recent study showed that a mutation targeting the C_L_ domain may also contribute to the aggregation behavior of the LC (221). Rottenacher et al. reported that a nonconservative valine to glycine replacement in the C_L_ of the amyloidogenic LC FOR005 influenced the amyloid pathway by destabilizing the fold of the C_L_ domain, which impacted the stability of the whole molecule. Consequently, the LC dimer interface was altered, weakening LC dimerization (221). They also showed that this change made the LC monomers more susceptible to proteolytic cleavage, resulting in the release of an isolated amyloidogenic V_L_ domain (221). This study is in line with the evidence indicating that the limiting step for LC fibrillogenesis is the dissociation of the native dimer into misfolded-prone monomers (222, 223, 240–242).

In summary, somatic mutations drive amyloid aggregation of the LCs through different mechanisms that directly or indirectly promote misfolding. It is believed that at least a fraction of the mutations involved in LC amyloid aggregation were positively selected due to their beneficial impact on antibody recognition (243). This contrasts with mutations associated with amyloid aggregation in other amyloid precursors, such as transthyretin, in which a biological advantage linked to the change has not been proven.

### 4.3 Impact of LC sequence heterogeneity on AL fibrillar structure

Cryo-EM structural model of four ex-vivo AL fibrils, all obtained from patients with cardiac AL amyloidosis, are currently available in the Protein Data Bank (PDB) (https://www.rcsb.org/). Two structures are of fibrils of Λ1 LCs, one identified with the PDB ID 6IC3 (233) and the other with the PDB ID 7NSL (234). The other two cryo-EM structures are of LCs of subgroups Λ3 (PDB ID 6Z1O and 6Z1I) (235) and Λ6 (PDB ID 3HUD) (232), respectively (**Figure 6**). This is still an insufficient number of structures to issue definitive conclusions regarding the relationship between LC sequence and AL fibril structure. However, they represent a huge step forward in our understanding regarding how the LCs assemble in the amyloid fibril, which forces stabilize the aggregate, and how somatic mutations influence it. In agreement with a previous ssNMR study (231), cryo-EM analyzes showed that AL fibrils are formed by stacking the V_L_ domains, refolded into a quasi-bidimensional, flattened structure, one on top of the other, with the parallel in-register arrangement of the β strands (232, 233). The fibrillar fold is totally different from the β-sandwich topology that characterizes the native V_L_, indicating that extensive conformational conversion must occur in some step before the LC assembles into AL fibrils *in vivo* (231). It is important to mention that only the V_L_ domain contributes to the β core of the fibril. Most of the V_L_ segments adopt a defined and stable conformation in the fibril´s β-core, while others, mostly the N- and C-terminals, but also some internal segments, are disordered and exposed to the solvent (**Figure 6**).

The intradomain disulfide bond Cys23-Cys88 is conserved in all reported AL fibril structures, indicative that LC amyloidogenesis occurs in an oxidative environment (235) (**Figure 6**). A structural motif common to all AL fibrils is the 180° relative rotation, centered on the Cys23-Cys88 disulfide bond, of the segments corresponding to the native β strands B and F. This rearrangement changes the relative orientation N-term-to-C-term from the parallel, characteristic of the native state, to antiparallel (233).

The primary force stabilizing the AL fibrils seems to be the network of H-bonds between the main chain peptide groups of adjacent monomers (**Figure 6**). Additionally, the amino acid side chains in the AL fibril´s core interdigitate each other in a tight complementary fashion, forming steric zipper-like unions (244, 245). Nonpolar amino acids tend to cluster and locate buried, while those with polar or charged side chains tend to be solvent exposed. Aromatic amino acids interact through intermolecular pi-stacking, a type of interaction that has been shown to contribute to the stability of both pathogenic and functional amyloids (78, 246, 247).

Apart from some common elements, Cryo-EM analysis confirmed that structural heterogeneity is the hallmark of the AL fibrils. The content of the β structure and the general topology of the fibrillar V_L_ were found to differ widely from one AL fibril to another (**Figure 6**). Fibril-specific salt bridges, which in some cases resulted from somatic mutations, seen to contribute significantly to the stability of the V_L_ fold in some AL fibrils (234, 248). Cryo-EM studies also suggest that N-glycosylation, one of the post-translational modifications (PTM) found in amyloidogenic LCs, may also contribute to the structural heterogeneity of AL fibrils (234). Radamaker et al. found that the AL fibril FOR001 was N-glycosylated at Asn18, a residue introduced by somatic mutation, (234). Cryo-EM analysis suggested that the carbohydrate moiety, which was exposed at the surface of the FOR001 fibrils, determined the unique fold adopted by FOR001 LC in the fibril (234) (**Figure 6**).

Studies conducted in the 1980s and 1990s suggested a link between glycosylation and the propensity of LCs to cause amyloidosis and kidney impairment (249–253). Subsequent studies based on the comparative analysis of amyloidogenic and non-amyloidogenic LC sequences showed a preponderance of the consensus glycosylation sequon (AsnXxxSer/Thr) in the FRs of amyloid LCs (254). Based on these analyses, the acquisition of an N-linked glycosylation site through somatic mutations was proposed by Fred J. Stevens as one of four structural risk factors identifying most fibril-forming κ LCs (255). The development of high-throughput procedures based on high-performance liquid chromatography-mass spectrometry (HPLC-MS) for the rapid analysis of LC glycosylation has greatly facilitated the investigation of the impact of glycosylation on LC amyloidogenesis (256, 257). In a recent study, Dispenzieri et al., provided strong evidence that monoclonal LC glycosylation is a potent risk factor for progression of monoclonal gammopathy of undetermined significance (MGUS) to AL amyloidosis, myeloma, and other PCDs (258). More recently, Nevone et al., found evidence for an N-glycosylation hot spot in amyloidogenic κ LCs that differentiates them from their non-amyloidogenic counterparts (259). They found that the majority of N-glycosylation sites in the pool of amyloidogenic κ LC analyzed consisted of the NFT sequon and were located in the FR3, particularly within the β-strand E. Of note, genetic analysis showed that somatic mutations in the context of progenitor glycosylation sites, rather than genomic variants, were responsible for the N-glycosylation hot spot identified in amyloidogenic κ LCs (259). The mechanisms by which glycosylation promotes LC amyloidogenesis are still poorly understood. This posttranslational modification was found to be more frequent in AL κ than in AL Λ LCs (256). It was initially suggested that it may increase the intrinsic aggregation potential of the LC by destabilizing its native fold, and/or decreasing its solubility (255). However, *in vitro* studies have shown that glycosylation can retard, rather than accelerate, LC aggregation kinetics (234, 260). Based on these findings, Radamaker et al. have proposed that glycosylation may promote amyloid deposition by increasing the resistance of AL fibrils to natural mechanisms that promote the removal of tissue protein aggregates, such as metalloprotease-mediated digestion (234). However, it cannot be ruled out that glycosylation may also promote AL deposition through mechanisms dependent on its impact on the properties of the LC native state.

The monoclonal LC involved in the amyloid deposits is unique for each AL amyloidosis patient, as are the internal structure and pathological properties of the fibrillar and non-fibrillar aggregates that this protein can generate. As stated throughout this review, this is a direct consequence of the genetic mechanisms that generate a diverse repertoire of polyclonal antibodies (143, 261). The LC’s unique structural and physicochemical properties, in interplay with the genetic background and biological momentum of the affected individual, result in a disease that, although named by practice purpose “AL amyloidosis”, is actually a patient-specific disorder (143). At the same time, it is logical to ask whether the AL fibrils formed by the same monoclonal LC in different organs of the same patient are also structurally different. In this regard, Ricagno et al. determined the cryo-EM structure of AL fibrils extracted from the kidney of an AL patient whose cardiac amyloid structure had previously been determined. It was found that the structure of the renal fibril was virtually identical to that reported for the cardiac fibril (262). This finding is highly relevant to understanding the role of the tissue microenvironment in AL fibril formation and may foster the development of fibril-targeted therapeutic approaches, such as those based on antibodies, for AL amyloidosis (263).

### 4.4 Light chain cardiotoxicity. Is it possible to predict it?

LCs are the amyloid precursor with the greatest sequence diversity, a property that translates into heterogeneity in the clinical course of patients with AL amyloidosis (143, 264). This characteristic, and the fact that in many patients the onset of the disease is insidious, frequently leads to delay in the diagnosis (265). Hence, a significant fraction of patients is diagnosed when they already present signs and symptoms of failure of vital organs, which reduces the therapeutic options and increases the risk of dying (266). The heart and kidneys are the most frequently affected organs, but it is cardiac amyloid infiltration that exerts the most direct impact on the patient’s survival (265, 267–269). This has stimulated research aimed at unraveling the mechanisms by which circulating monoclonal LC and its AL fibrils damage cardiomyocytes. Studies carried out in cell culture demonstrated that soluble LCs obtained from AL patients, but not their non-pathological counterpart, impair the cardiomyocyte contractile function by oxidative stress, inducing apoptosis and inflammatory changes (270–273). The mechanism by which synthetic AL fibrils exert cytotoxicity in cultured cardiomyocytes has also been investigated. Marin-Argany et al. investigated the mechanism of internalization of the amyloidogenic κ1 LC AL-09 into human AC16 cardiomyocytes and found that both the soluble protein and its fibrillar aggregates were internalized *via* micropinocytosis (274). They found that the external fibrillar aggregates interacted with cell membranes and recruited soluble LCs, promoting their aggregation through seeding. While both soluble LC and fibrils were cytotoxic, fibrils were significantly more so and exerted their toxic effect through different mechanisms than soluble LC. It was shown that soluble protein activated the caspases, suggestive of apoptosis induction (274).

Studies carried out in cell cultures, such as those cited here, are well suited to investigate, under controlled conditions, the complex chain of events that result from the interaction of an amyloidogenic LC, either in the soluble or fibrillar state, with cells. However, this approach fails to provide information regarding the mechanisms of cell damage in the physically restricting 3D structure of the tissue. Radamaker et al. used scanning electron microscopy to determine how AL fibrils interact with cardiomyocytes in an ex-vivo tissue sample of AL cardiac amyloidosis (234). They found that the deposits of AL fibrils infiltrated and disrupted the ordered structure of the cardiomyocytes; the fibrils interacted with the surface of the cardiomyocytes, mainly through the tips of the fibrils, causing in some cases deformations in the cytoplasmic membrane (234). Such physical perturbation likely impaired the contractile function of cardiomyocytes, causing heart failure. Based on this finding, the authors deduced that promoting the remotion of the AL deposits may help restore normal cardiac function (234).

Cardiotoxic LCs can also impair heart function by modulating the expression of proteins that mediate cardiomyocyte damage by indirect mechanisms. Guan et al. reported that mammalian stanniocalcin1 (STC1) expression is elevated in the cardiac tissue of patients with AL cardiomyopathy and that this protein is induced in isolated cardiomyocytes in response to amyloidogenic LC, but not to non-amyloidogenic LC (275). They showed that STC1 overexpression *in vitro* recapitulated the pathophysiology of AL LC mediated cardiotoxicity, with increased ROS production, contractile dysfunction, and cell death. They corroborated these findings in a zebrafish model (276), where it was found that overexpression of STC1 resulted in significant cardiac dysfunction and cell death, while genetic silencing prevented AL LC-induced cardiotoxicity and protected the animal from an early death. Hence, the authors concluded that STC1 as a critical determinant of AL LC cardiotoxicity (275).

A proteomic-based study showed that amyloidogenic cardiotoxic LCs interact *in vitro* with specific intracellular proteins involved in viability and metabolism in human cardiac fibroblasts (hCFs) (277). Cardiotoxic LCs, but not non-amyloidogenic non-cardiotoxic controls colocalized with mitochondria and spatially associated with specific cell components such as mitochondrial optic atrophy 1-like protein and peroxisomal acyl-coenzyme A oxidase 1. Additionally, cardiotoxic LC-treated hCFs displayed mitochondrial ultrastructural changes, supporting mitochondrial involvement (277).

Having an animal model that recapitulates the clinical evolution and anatomopathological changes of cardiac amyloidosis would give a great boost to research on this grave complication. In 1992, Solomon et al. reported the successful experimental induction of human AL amyloid deposits in mice by the repeated injection into the animal of Bence Jones proteins obtained from two patients with AL amyloidosis (278). Although this approach was able to produce the characteristic histopathologic lesions of the disease, it suffers from several limitations in terms of reproducibility and ability to replicate the natural course of AL amyloidosis (279). Therefore, several groups have focused their work on the generation of AL amyloidosis models in transgenic mice (280, 281). Although the transgenic mice produced the amyloidogenic LC at serum concentrations equivalent to or higher than the average concentration seen in patients with AL amyloidosis, spontaneous amyloid deposition has not been observed or occurred in small amounts (280, 281). Christophe Sirac’s group has implemented an alternative approach that comprises the intraperitoneal injection of preformed homologous amyloid fibrils in the transgenic mice, as a way of promoting tissue amyloid deposition by seeding. This strategy resulted in amyloid deposition starting at 1-month post-injection, especially in the heart, spleen, liver and, to a lesser extent, in the kidney (279). Although it represents an important step forward, this model still does not fully reproduce the natural progression of the disease, since it suffers from little accumulation of tissue amyloid deposits and slow progression (279, 282). It is not clear what makes mice so resistant to the spontaneous amyloid deposition of LCs demonstrably amyloidogenic in humans. It has been speculated that mice may have developed more effective mechanisms of protein aggregates clearance or a better chaperoning of misfolded proteins than in humans (279).

Other approaches have been explored by other laboratories, such as those based on zebrafish (276, 283) and C. elegans (284, 285). Using a zebrafish model, Guan et al. determined that dysregulation of autophagic flux is critical for mediating amyloidogenic LC cardiac proteotoxicity, an effect that they were able to revert by restoration of autophagic flux by pharmacological intervention using rapamycin (286). They identified impaired lysosomal function as the major cause of defective autophagy and amyloidogenic LC-induced cardiotoxicity (286).

The use of C. elegans as a model of AL cardiac amyloidosis relies in the knowledge that the worm’s pharynx is an “ancestral heart” with the additional ability to recognize stressor compounds (284, 285). Using pharyngeal pumping as a surrogate for cardiac function, Diomede et al. demonstrated that cardiotoxic amyloidogenic LCs directly affect pharyngeal activity. The toxic effect was associated with the release of reactive oxygen species since the use of antioxidant agents restored pharyngeal activity (284).

The same C. elegans model was recently used by Maritan et al. to investigate the role of sequence on LC cardiotoxicity (287). They hypothesized that LC conformational flexibility contributes to cardiotoxic properties. To test this hypothesis, the amino acid sequence of the highly cardiotoxic LC H6 was rationally engineered by introducing three residue mutations, designed to reduce the dynamics of its native state (287). The resulting mutant (mH6) was less toxic than its parent H6 to human cardiac fibroblasts and C. elegans. The comparative structural and biophysical study of H6 and mH6 indicated that H6 featured a poorly cooperative fold, higher flexibility, and kinetic instability, and a higher dynamic state in its native fold (287). This study suggests a close relationship between the general conformational properties of the LC native fold and its ability to cause cardiotoxicity.

Understanding what makes a monoclonal LC cardiotoxic would allow calculating the risk of cardiac damage in newly diagnosed AL amyloidosis patients, a huge step forward in the effort to improve the prognosis and treatment of AL patients. Due to the complexity of this task derived from the high sequence variability of LCs, computational algorithms to predict cardiotoxicity based on the computable properties of LCs are considered a promising strategy. Several algorithms have been developed to predict amyloidogenesis in proteins (288, 289), but to the best of our knowledge, only one algorithm, LICTOR (Λ-LIght-Chain TOxicity predictoR), a machine learning-based tool, was designed with the specific goal of predicting Λ-LC proteotoxicity in mind (290). LICTOR predicts AL Λ-LC toxicity based on the distribution of somatic mutations acquired during clonal selection. This tool achieved a specificity and sensitivity of 0.82 and 0.76, respectively. The calculated area under the receiver operating characteristic curve (AUC) was 0.87. Moreover, LICTOR achieved a prediction accuracy of 83% on an independent set of 12 Λ-LCs sequences with known clinical phenotypes. Using this algorithm, the authors were able to identify two somatic mutations predicted to be responsible for the toxic phenotype, and abolished this property, as tested in the C. elegans model, by reverting the changes to the germline-specific residues (290).

Undoubtedly, LICTOR represents an important advance in the effort to develop the ability to predict the cardiotoxicity of LC, which, if achieved, would open the door to new forms of diagnosis, prognosis, and treatment of AL amyloidosis patients. According to the report of Kourelis et al., ∼65% of patients with systemic AL amyloidosis will eventually develop cardiac involvement, which highlights the need for effective tools for predicting this grave complication (170). At the same time, it is important to point out that acquiring the ability to predict the cardiotoxic potential of a monoclonal LC solves only part of the problem. There are challenges that would have to be undertaken before a tool like LICTOR finds its place in the diagnosis and therapeutic management of AL amyloidosis. Predictors of LC cardiotoxicity may help to identify patients with circulating monoclonal LC at risk of cardiac amyloidosis, which could be a candidate for more aggressive treatments to eliminate the pro-amyloidogenic plasma cell clone. However, the prediction of amyloidogenicity requires the sequence of the LC, which is not easily obtained, especially in the absence of a diagnosis of amyloidosis. Moreover, currently, many patients are still diagnosed when the signs and symptoms of organ damage, including heart failure, are already present (150, 291, 292). Improving our ability to diagnose AL patients at the earliest stages of the disease would increase the relevance of this type of predictive tool.

### 4.5 Somatic mutations in renal amyloidosis and the role of mesangial cells

Studies conducted *in vitro* demonstrated that LC amyloid aggregation can be accomplished without the participation of cells (183, 213). These studies critically contributed to our current understanding of the mechanism of LC misfolding and amyloid aggregation and, moreover, showed that the potential of an LC to form amyloid fibrils is determined by its sequence (152, 160, 172, 173, 198). As a rule, *in vitro* aggregation experiments are performed with the sole presence of the monoclonal LC dissolved in a buffer solution (213). This is a situation far distant from reality in humans where the circulating monoclonal LC must interact with both cells and components of the extracellular matrix in the formation of amyloid fibrils. The evidence supports the notion that it is through the interplay between the physicochemical and structural properties of the LC and factors of the tissue microenvironment that the amyloidogenic potential of a monoclonal LC translates into amyloid deposition (78). Therefore, unraveling the role of the tissue components in LC amyloidogenesis is an inescapable task in the effort to understand the pathogenesis of AL amyloidosis. Studies conducted in the last two decades provided abundant evidence of the involvement of mesangial cells in AL deposition in the renal glomerulus and pericytes/smooth muscle cells in the vasculature (293–298). It is now known that the circulating monoclonal LC interacts with different cell types and the consequences of such interaction depend on two main factors: 1) the structural and physicochemical characteristics of the LC, which are dictated by the LC sequence, and 2) the ability of certain cell types to internalize the LC and promote its aggregation into amyloid fibrils in the mature lysosomal compartment (297, 298).

In healthy individuals, the kidneys catabolize ∼50 mg per day of polyclonal free LCs, which originate from the excess production of LC over the HC that occurs in the normal B cells (299, 300). Once filtered in the glomerulus and delivered to the proximal tubules, the polyclonal LCs are avidly endocytosed by the cubilin-megalin receptor, a unique receptor for low molecular weight proteins located at the microvillous surface of the proximal tubular cells (301–303). Then, the endosomes in the proximal tubules catabolize the internalized LCs, returning the amino acids to circulation. Due to the low concentration and structural heterogeneity of polyclonal free LCs circulating in the blood of normal individuals, tissue deposition rarely occurs (304).

As mentioned before, in a fraction of patients with plasma cell dyscrasias, the abnormally proliferating plasma cell clone produces the monoclonal LC in large excess, which is secreted in a free state to the bloodstream and reaches the kidneys. From 50% to 70% of patients with a circulating monoclonal LC have clinical evidence of renal damage, indicating that it is nephrotoxic (305). Approximately 70% of the nephrotoxic mLC cause tubular injury and are referred to as tubulopathic LCs (TLCs). The remaining 30% are associated with glomerular alterations and are termed glomerulopathic LCs (GLCs). Only a few monoclonal LCs are both TLC and GLC (297, 298).

GLCs produce two diametrically opposite diseases in terms of their renal pathological expressions: AL amyloidosis and LC deposition disease (LCDD) (**Figures 3** and **4**). As was mentioned previously, in AL amyloidosis, the pathogenic monoclonal LC, most commonly of Λ-type, deposits in the extracellular space in the form of randomly arranged nonbranching fibrils with a diameter of 10-12 nm (306). As renal amyloidosis progresses, the mesangial matrix is destroyed and replaced by AL fibrils (**Figure 4**). The destruction of the native mesangial matrix occurs because of the direct activation of matrix metalloproteinases (MMPs). In contrast, in LCDD, the monoclonal LC, most commonly of κ type, deposits in the mesangium and along glomerular and tubular basement membranes. Electron microscopy analysis shows mesangial and inner glomerular and outer tubular basement membrane deposits of extracellular punctate, powdery, ground-pepper-like deposits (307) (**Figure 3**). Activation of TGF-β leads to the formation of mesangial nodules and deposition of tenascin replacing the predominant collagen IV matrix that is present in the normal mesangium (297).

The two diseases have been reproduced in experimental platforms permitting crucial evaluation of the step-by-step mechanisms involved. Three experimental platforms, with distinct levels of complexity, have been used to study interactions between GLCs and MCs. The first uses MCs in culture with or without a matrix, which are incubated with GLC. The second is an ex-vivo platform in which an explanted rat or mice kidneys is perfused with the GLC through the renal artery. The third is an *in-vivo* animal model that involves penile injections of LCs in mice (297). These experimental platforms have provided evidence that the interaction of the GLCs with MCs involves a receptor: SORL1 (298). The interaction GLC-MC triggers a chain of molecular events that determine the pathophysiological response and fate of the cell. It is important to emphasize that, although both the LCs from patients with AL or LCDD interact with the same membrane receptor on MCs, the cellular response, and ultimate pathological changes that they promote differ significantly (298).

**Figure 7:**
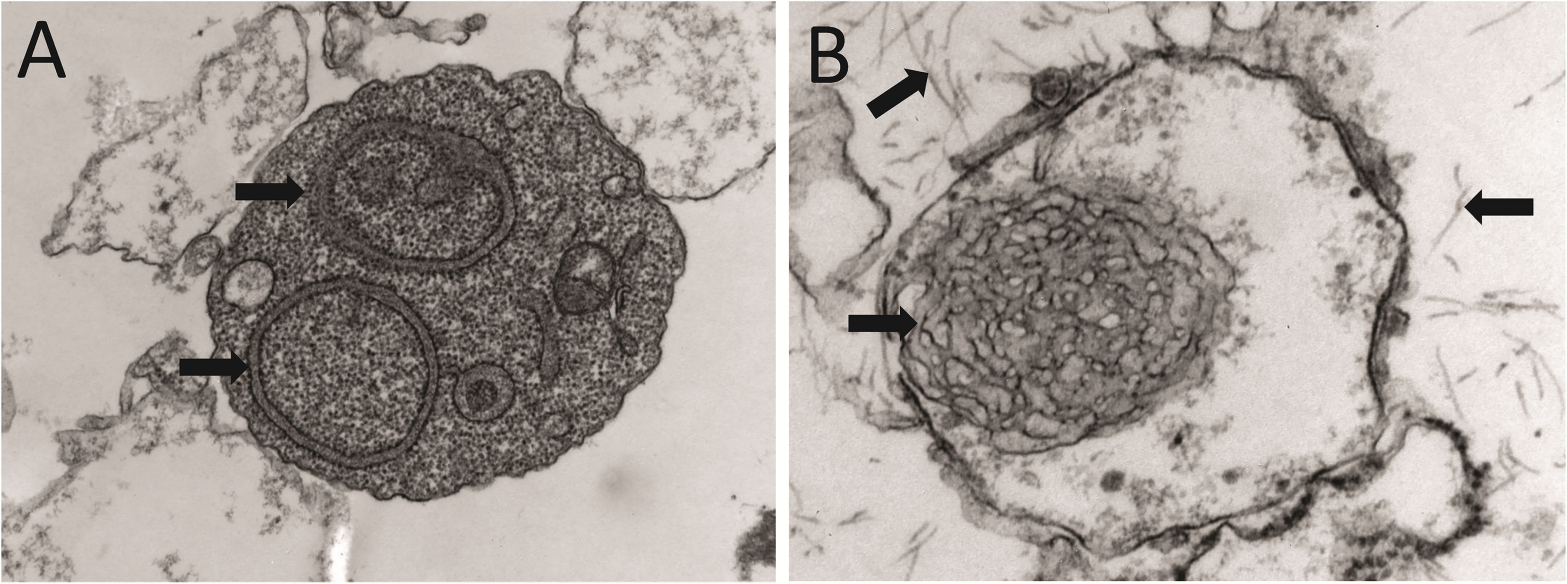
Intralisosomal processing and fibrillar aggregation of an amyloidogenic LC in human mesangial cells. EM micrographs of lysosomes obtained from cultured human mesangial cells that were incubated with an amyloidogenic Λ LC purified from the urine of a patient with AL amyloidosis. After **(A)** 30 min and **(B)** 2 h, the cells were harvested, and the lysosomal fraction was obtained by density gradient ultracentrifugation. Note in **(A)** the intralysosomal amyloid fibrils arranged in perfect circles (black arrows). In **(B)**, the amyloid fibrils show a less orderly arrangement (black arrow inside the lysosome). Note some amyloid fibrils that appear to have escaped from the lysosome (black arrows outside the lysosome).

When an AL LC interacts with mesangial cells at the cell surfaces, signals are activated, and the LC is endocytosed into the cells and routed to the mature lysosomal system for processing (293, 294, 296). Rab proteins participate in the migration of the monoclonal LC from the mesangial cell surface to the lysosomes. Once in the lysosomes, the stringent low pH and potentially the presence of lysosomal proteases promote LC misfolding and aggregation into amyloid fibrils (298, 308) (**Figure 7**). The last step involves the exocytosis of the fibrils into the extracellular compartment. Two factors, C-fos and NF-kB, control the mesangial cell activities leading to amyloid fibril formation (309). They are instrumental in fostering cellular activities that control amyloidogenesis. C-fos is necessary for the phenotypic transformation of mesangial cells into a macrophage phenotype, a crucial event that needs to occur before mesangial cells can engage in active amyloid formation (294, 296). The same is true about vasculature where the AL LC interacts with pericytes/smooth muscle cells to produce amyloid, through a process that is amazingly like that taking place in the renal mesangium (295). In contrast, the incubation of MC with LCDD LCs appears to activate different signaling pathways, since LC internalization does not occur. Moreover, treated MCs undergo a completely different phenotypic transformation, in this case toward a myofibroblasts phenotype, acquiring enhanced protein synthesis function (294).

Overall, these studies strongly suggest that the physicochemical and structural properties of the monoclonal LC influence how MCs respond to the LC interaction. Therefore, both the IGV_L_ gene segment encoding the monoclonal LC and somatic mutations can be anticipated to modulate pathogenic events triggered by GLC-MC interaction, such as cell-mediated LC amyloidogenesis. Determining how the LC sequence modulates the pathogenic response of MCs will significantly expand our understanding of the structural bases of AL amyloidosis and LCDD and may pave the way for developing more effective therapeutic approaches for these devastating diseases.

## 5 Conclusion

The development of an adaptive immune system in jawed vertebrates gave them the ability to produce antibodies and T-cell receptors specific to virtually any foreign molecule. Such highly diverse repertoire of antibody/T-cell receptor is generated by two main mechanisms, V(D)J gene segment recombination and SHM. Given the very molecular nature of these mechanisms, there is a latent risk of genomic instability that may result in the malignant transformation of the cell. Moreover, the insertion of multiple mutations in the variable region of the antibody LCs by somatic hypermutation can promote misfolding and tissue deposition of this protein if it is secreted in a free state. Important advances have been made in understanding the molecular mechanisms that generate diversity in the adaptive immune system. In the same way, we now know more regarding the causes and mechanisms of the pathological aggregation of LCs, which opens new opportunities for better diagnosis and treatment of patients with AL amyloidosis, LCDD, myeloma (cast) nephropathy, and other disorders caused by LC deposition. However, given the extremely high antibody diversity generated by the aforementioned mechanisms, understanding what governs the pathogenic properties of a LC remains a major challenge.

## Author contributions

LP-Y and GH determined the general theme, objectives, and structure of the review, and designed and prepared the figures. All authors contributed to the writing, revision, and correction of the manuscript. All authors contributed to the article and approved the submitted version.
